# Socioeconomic Inequalities in Lung Cancer Treatment: Systematic Review and Meta-Analysis

**DOI:** 10.1371/journal.pmed.1001376

**Published:** 2013-02-05

**Authors:** Lynne F. Forrest, Jean Adams, Helen Wareham, Greg Rubin, Martin White

**Affiliations:** 1 Fuse, UKCRC Centre for Translational Research in Public Health, Institute of Health & Society, Newcastle University, Newcastle upon Tyne, United Kingdom; 2 Wolfson Research Institute, Durham University, Queen's Campus, Stockton on Tees, United Kingdom; World Health Organization, Switzerland

## Abstract

**Background:**

Intervention-generated inequalities are unintended variations in outcome that result from the organisation and delivery of health interventions. Socioeconomic inequalities in treatment may occur for some common cancers. Although the incidence and outcome of lung cancer varies with socioeconomic position (SEP), it is not known whether socioeconomic inequalities in treatment occur and how these might affect mortality. We conducted a systematic review and meta-analysis of existing research on socioeconomic inequalities in receipt of treatment for lung cancer.

**Methods and Findings:**

MEDLINE, EMBASE, and Scopus were searched up to September 2012 for cohort studies of participants with a primary diagnosis of lung cancer (ICD10 C33 or C34), where the outcome was receipt of treatment (rates or odds of receiving treatment) and where the outcome was reported by a measure of SEP. Forty-six papers met the inclusion criteria, and 23 of these papers were included in meta-analysis. Socioeconomic inequalities in receipt of lung cancer treatment were observed. Lower SEP was associated with a reduced likelihood of receiving any treatment (odds ratio [OR] = 0.79 [95% CI 0.73 to 0.86], p<0.001), surgery (OR = 0.68 [CI 0.63 to 0.75], p<0.001) and chemotherapy (OR = 0.82 [95% CI 0.72 to 0.93], p = 0.003), but not radiotherapy (OR = 0.99 [95% CI 0.86 to 1.14], p = 0.89), for lung cancer. The association remained when stage was taken into account for receipt of surgery, and was found in both universal and non-universal health care systems.

**Conclusions:**

Patients with lung cancer living in more socioeconomically deprived circumstances are less likely to receive any type of treatment, surgery, and chemotherapy. These inequalities cannot be accounted for by socioeconomic differences in stage at presentation or by differences in health care system. Further investigation is required to determine the patient, tumour, clinician, and system factors that may contribute to socioeconomic inequalities in receipt of lung cancer treatment.

*Please see later in the article for the Editors' Summary*

## Abstract 2

In a systematic review and meta-analysis, Lynne Forrest and colleagues find that patients with lung cancer who are more socioeconomically deprived are less likely to receive surgical treatment, chemotherapy, or any type of treatment combined, compared with patients who are more socioeconomically well off, regardless of cancer stage or type of health care system.

## Editors' Summary

**Background:**

Lung cancer is the most commonly occurring cancer worldwide and the commonest cause of cancer-related death. Like all cancers, lung cancer occurs when cells begin to grow uncontrollably because of changes in their genes. The most common trigger for these changes in lung cancer is exposure to cigarette smoke. Most cases of lung cancer are non-small cell lung cancer, the treatment for which depends on the “stage” of the disease when it is detected. Stage I tumors, which are confined to the lung, can be removed surgically. Stage II tumors, which have spread to nearby lymph nodes, are usually treated with surgery plus chemotherapy or radiotherapy. For more advanced tumors, which have spread throughout the chest (stage III) or throughout the body (stage IV), surgery generally does not help to slow tumor growth and the cancer is treated with chemotherapy and radiotherapy. Small cell lung cancer, the other main type of lung cancer, is nearly always treated with chemotherapy and radiotherapy but sometimes with surgery as well. Overall, because most lung cancers are not detected until they are quite advanced, less than 10% of people diagnosed with lung cancer survive for 5 years.

**Why Was This Study Done?:**

As with many other cancers, socioeconomic inequalities have been reported for both the incidence of and the survival from lung cancer in several countries. It is thought that the incidence of lung cancer is higher among people of lower socioeconomic position than among wealthier people, in part because smoking rates are higher in poorer populations. Similarly, it has been suggested that survival is worse among poorer people because they tend to present with more advanced disease, which has a worse prognosis (predicted outcome) than early disease. But do socioeconomic inequalities in treatment exist for lung cancer and, if they do, could these inequalities contribute to the poor survival rates among populations of lower socioeconomic position? In this systematic review and meta-analysis, the researchers investigate the first of these questions. A systematic review uses predefined criteria to identify all the research on a given topic; a meta-analysis is a statistical approach that combines the results of several studies.

**What Did the Researchers Do and Find?:**

The researchers identified 46 published papers that studied people with lung cancer in whom receipt of treatment was reported in terms of an indicator of socioeconomic position, such as a measure of income or deprivation. Twenty-three of these papers were suitable for inclusion in a meta-analysis. Lower socioeconomic position was associated with a reduced likelihood of receiving any treatment. Specifically, the odds ratio (chance) of people in the lowest socioeconomic group receiving any treatment was 0.79 compared to people in the highest socioeconomic group. Lower socioeconomic position was also associated with a reduced chance of receiving surgery (OR = 0.68) and chemotherapy (OR = 0.82), but not radiotherapy. The association between socioeconomic position and surgery remained after taking cancer stage into account. That is, when receipt of surgery was examined in early-stage patients only, low socioeconomic position remained associated with reduced likelihood of surgery. Notably, the association between socioeconomic position and receipt of treatment was similar in studies undertaken in countries where health care is free at the point of service for everyone (for example, the UK) and in countries with primarily private insurance health care systems (for example, the US).

**What Do These Findings Mean?:**

These findings suggest that patients in more socioeconomically deprived circumstances are less likely to receive any type of treatment, surgery, and chemotherapy (but not radiotherapy) for lung cancer than people who are less socioeconomically deprived. Importantly, these inequalities cannot be explained by socioeconomic differences in stage at presentation or by differences in health care system. The accuracy of these findings may be affected by several factors. For example, it is possible that only studies that found an association between socioeconomic position and receipt of treatment have been published (publication bias). Moreover, the studies identified did not include information regarding patient preferences, which could help explain at least some of the differences. Nevertheless, these results do suggest that socioeconomic inequalities in receipt of treatment may exacerbate socioeconomic inequalities in the incidence of lung cancer and may contribute to the observed poorer outcomes in lower socioeconomic position groups. Further research is needed to determine the system and patient factors that contribute to socioeconomic inequalities in lung cancer treatment before clear recommendations for changes to policy and practice can be made.

**Additional Information:**

Please access these Web sites via the online version of this summary at http://dx.doi.org/10.1371/journal.pmed.1001376.

- The US National Cancer Institute provides information about all aspects of lung cancer for patients and health care professionals (in English and Spanish); a monograph entitled Area Socioeconomic Variations in U. S. Cancer Incidence, Mortality, Stage, Treatment, and Survival, 19751999 is available
- Cancer Research UK also provides detailed information about lung cancer and links to other resources, such as a policy statement on socioeconomic inequalities in cancer and a monograph detailing cancer and health inequalities in the UK
- The UK National Health Service Choices website has a page on lung cancer that includes personal stories about diagnosis and treatment
- MedlinePlus provides links to other US sources of information about lung cancer (in English and Spanish)

## Introduction

Lung cancer is the most commonly occurring cancer worldwide. In the USA and the UK it is the second most incident cancer [1],[2], as well as the most common cause of cancer mortality [2],[3]. Survival differs internationally. In the UK, fewer than 10% of those diagnosed with lung cancer survive for 5 years [3],[4], with higher survival rates found in Nordic countries [4],[5], the USA [2],[5], Australia, and Canada [4].

Lung cancers are classified into small cell (SCLC) and non-small cell (NSCLC) lung cancers. NSCLC is more common than SCLC and has a better survival rate [6]. National Institute for Health and Clinical Excellence (NICE) guidelines recommend radical surgery for stage I or II NSCLC [6]. Chemotherapy and radiotherapy are recommended for later-stage NSCLC patients and are the treatments of choice for SCLC [6]. Treatment intervention with surgery, chemotherapy, or radiotherapy has been shown to improve lung cancer survival [6].

Socioeconomic inequalities in incidence of, and survival from, the majority of cancers have been reported [1],[3],[7]. A recent non-systematic review revealed socioeconomic inequalities in receipt of treatment for colorectal cancer [8], and it has been suggested that socioeconomic differences in access to treatment might at least partially explain socioeconomic differences in survival [9]. Unintended variations in outcome that result from the way that health interventions are organised and delivered have been described as intervention-generated inequalities [10].

Incidence of lung cancer is higher [1],[11], and survival poorer [7], in the most deprived patient groups. However, it is not known whether socioeconomic inequalities in receipt of treatment exist for lung cancer and, if so, what contribution they make to overall socioeconomic inequalities in outcome. In order to explore the first of these questions, we undertook a systematic review and meta-analysis of cohort studies examining the association between socioeconomic position (SEP) and receipt of lung cancer treatment.

## Methods

A protocol (see Text S2) was developed and systematic methods were used to identify relevant studies, assess study eligibility for inclusion, and evaluate study quality. The review is reported according to the Preferred Reporting Items for Systematic Reviews and Meta-Analyses (PRISMA) guidelines [12] (see Text S1 for PRISMA checklist).

### Literature Search

The online databases of MEDLINE and EMBASE were searched up to September 2012 (see Table S1 for full search strategies). No language restriction was applied. A search of Scopus uncovered no further papers. Additional studies were identified by reviewing the reference lists of all included studies and by using a forward citation search to identify more recent studies that had cited included studies. EndNote X5 software was used to manage the references.

### Study Eligibility

Studies that met the following criteria were included in the review: primary, cohort studies of participants with a primary diagnosis of lung cancer (ICD10 C33 or C34) reported separately from other cancers; published in a peer-reviewed journal; where at least one reported outcome was receipt of treatment (measured by rates or odds of receiving treatment); and where receipt of this outcome was reported by a measure of SEP. Any curative or palliative treatment for lung cancer including surgery, chemotherapy, and radiotherapy was included.

Studies where SEP was included as a descriptive variable or confounder, but where outcomes for receipt of treatment by SEP were not presented, were not eligible for inclusion, but the authors were contacted to determine whether relevant data were available that might allow for inclusion in the review.

Studies where multivariable analysis was conducted (and included control for a minimum of age and sex as confounders); receipt of treatment was compared to not receiving treatment; odds ratios (ORs) and 95% confidence intervals (CIs) of receipt of treatment in low compared to high SEP were calculated; and SEP was not further stratified by another variable, were considered suitable for inclusion in meta-analysis.

Acceptable measures of SEP were: area-based indices of deprivation (e.g., Index of Multiple Deprivation [IMD], Townsend Score, Carstairs Index); and area or individual measures of income, poverty, or education level.

Multiple papers using the same or overlapping study data were included. Sensitivity analyses were conducted including all eligible papers and using different combinations of included papers, but only data from the better quality or more detailed paper in each overlapping study group were included in the final meta-analyses. Sensitivity meta-analyses are included in the supplemental material.

### Study Selection and Data Extraction

Studies obtained from the database searches were independently assessed by two researchers (LFF and HW) in three phases: title, abstract, and full paper screening. Any disagreements at any of the screening stages were resolved by discussion between the two researchers in the first instance and with a third reviewer (JA) if agreement could not be reached. Data extraction was carried out by LFF using an Access database pro-forma developed for this purpose, and double-checked by HW.

There is evidence to suggest that health insurance status is an important factor relating to access to lung cancer care in countries such as the USA that rely on insurance-based health care systems [13]. Insurance status is less relevant and rarely measured in most other countries. Therefore, three analytical categories were developed a priori: studies conducted in a universal health care system (UHCS), free at the point of access (similar to the UK); studies conducted in countries with primarily private insurance health care systems (non-UHCS, similar to the USA) [14]; and studies conducted in countries with social insurance health care systems (similar to many European countries). No studies were identified that fell into the third category.

### Study Quality

A study quality tool, adapted from existing quality tools [15],[16], was used to divide studies into six quality categories, with 1 being the lowest, and 6 the highest, quality (see Text S3). Quality assessment was carried out by LFF and checked by HW.

Cohort studies reporting only univariable analysis are of lower quality in terms of their ability to control for confounding. Only studies conducting multivariable analysis (quality scores 3–6) were included in the meta-analysis. All studies that met the inclusion criteria were analysed in the narrative synthesis.

### Statistical Analysis

Trends in receipt of treatment across SEP groups were described in the narrative analysis of all studies that met the inclusion criteria.

Meta-analysis of eligible studies was undertaken using Cochrane Collaboration Review Manager 5.1. Natural logs of the ORs and their standard errors (SEs) were calculated for use in forest plots. Random-effects meta-analysis of the odds of treatment in the lowest compared to the highest SEP group was conducted. Where a study reported the most deprived class as the comparator, reverse ORs were calculated. Studies that presented a single OR as either an OR for a one unit increase in deprivation score or incremental quintile increase in income were not included.

Subgroup analyses by treatment type and health care system were conducted. In meta-analyses where a “substantial” percentage [17] of the variability appeared to be due to the heterogeneity of the studies rather than to chance, further subgroup analyses by stage, histology, and quality score were conducted, where appropriate, in order to examine potential sources of heterogeneity. A funnel plot was used to assess potential publication bias.

## Results

### Included Papers/Studies

**Figure 1:**
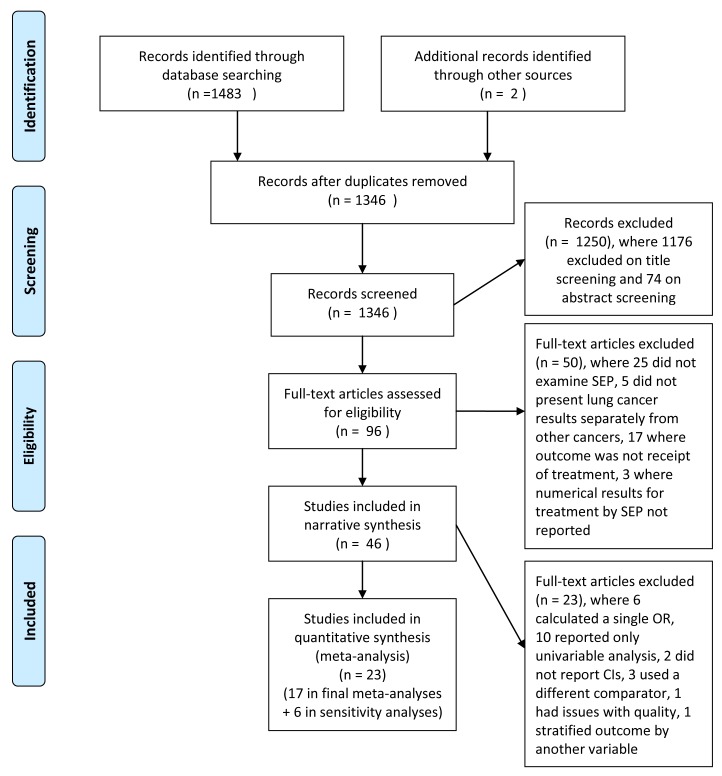
Flow diagram of study selection and exclusion. CI, confidence interval; SEP, socioeconomic position.

A total of 46 papers met the inclusion criteria and were included in the review (see the PRISMA flow diagram [Figure 1]). Twenty-eight papers were from UHCS countries (Tables 1 and 2). Of these, 19 UK papers examined 13 study populations, although as these included national and regional populations from different sources, there was some further population overlap. One UK paper also compared treatment in Scotland and Canada [18]. A further nine papers from Canada (2), Sweden (1), Australia (1), Italy (1), France (1), and New Zealand (3) were included. The three New Zealand papers all examined the same population.

**Table 1:** Characteristics of included studies potentially suitable for meta-analysis (universal health care systems).

Paper	Country of Study	Data Source (s)	Population Included	Years of Diagnosis	Measure of SEP	No. of SEP groups	Treatment given within	Age Range	Confounders Controlled For:					Quality Score
									Age	Sex	Stage	Histology	Other	
Berglund et al, 2010 [19]	Sweden	Regional Lung Cancer Register (RLCR) - Sweden, Cause of Death Register and LISA (insurance and demographics)	Uppsala/Orebro region in central Sweden	1996–2004	Education level^a^	3	NR	30+	Yes	Yes	Yes	Yes	Performance status, year of diagnosis, smoking status	6
Berglund et al, 2012 [22]	England	Thames Cancer Registry, HES, LUCADA	South-east England	2006–2008	IMD 2007 income domain	5	NR	0–80+	Yes	Yes	Yes	Yes	Co-morbidity	6
Campbell et al, 2002 [35]	Scotland	Scottish Cancer Registry and hospital case notes	Random sample from North/NE Scotland (with hospital record)	1995–1996	Carstairs Index	5	12 months	NR	Yes	Yes	Yes	Yes	Health board, distance to cancer centre, mode of admission	5
Crawford et al, 2009 [36]	England	Northern and Yorkshire Cancer Registry and Information Service (NYCRIS)	Northern and Yorkshire region	1994–2002	IMD 2004 (access to services domain removed)	4	6 months	NR	Yes	Yes	No	Yes	Travel time (but overall results not stratified by travel time used here). Histology not included in receipt of any treatment analysis.	4
Erridge et al, 2002 [37]	Scotland	Scottish Cancer Registry and medical records	Scotland (with hospital record)	1995	Carstairs Index	5	6 months	<60– 80+	Yes	Yes	Yes	Yes	Health board (not inc in receipt of radiotherapy), diagnosis by specialist, management by oncologist	6
Erridge et al, 2009 [18]	Scotland/Canada	Scottish Cancer Registry and medical records; British Columbia Cancer Registry	Scotland/British Columbia	1995	Carstairs Index/average household income	2	6 months	<60– 80+	Yes	Yes	Yes	Yes	Travel time, CT scan	4
Gregor et al, 2001 [38]	Scotland	Scottish Cancer Registry and medical records	Scotland (with hospital record)	1995	Carstairs Index	5	6 months	<60–80+	Yes	Yes	Yes	Yes	Referral to specialist within 6 months of diagnosis	6
Jack et al, 2003 [39]	England	Thames Cancer Registry	South-east England	1995–1999	Townsend (median score per health authority)	Contin-uous^b^	NR	<35–85+	Yes	Yes	Yes	Yes	First hospital visited is a radiotherapy centre, basis of diagnosis, incidence. Health authority/hospital used as 2^nd^ level in multi-level model.	4
Jack et al, 2006 [40]	England	Thames Cancer Registry and medical records	South-east London (with hospital record)	1998	IMD 2000	5	6 months	<55–85+	Yes	Yes	Yes	Yes	Consultant specialty, basis of diagnosis (hospital, number of symptoms in some analyses)	6
Jones et al,2008 [41]	England	Northern and Yorkshire Cancer Registry and Information Service (NYCRIS)	Northern and Yorkshire region	1994–2002	IMD 2004 (access to services domain removed)	Contin-uous^c^	NR	NR	Yes	Yes	No	Yes	Travel time to hospital	4
Mahmud et al, 2003 [42]	Ireland	National Cancer Registry of Ireland (NCRI)	Republic of Ireland	1994–1998	SAHRU area-based material deprivation index	3	6 months	15–80+	Yes	Yes	No	Yes	Health board, year of diagnosis	4/2^d^
McMahon et al, 2011 [43]	England	Eastern Cancer Registry and Information Centre (ECRIC)	East of England	1995–2006	IMD 2004 (access to services domain removed)	5	NR	<60–80+	Yes	Yes	No	Yes	Year of diagnosis	4
Pollock &Vickers, 1998 [44]	England	HES FCEs	North/South Thames (admitted to hospital)	1992–1995	Townsend	10	NR	<100	Yes	Yes	No	No	Hospital, mode of admission	3
Raine et al, 2010 [45]	England	HES FCEs	England (admitted to hospital)	1999–2006	IMD	5	NR	50– 90+	Yes	Yes	No	No	Trust, year of admission, mode of admission	3
Riaz et al, 2012 [34]	England	NCIN/UKACR cancer registries	England	2004–2006	IMD 2004	5	NR	0– 85+	Yes	Yes	No	No	Government Office Region	4
Rich et al, 2011(1) [46]	England	LUCADA supplied by 157 NHS trusts	England	2004–2007	Townsend	5	NR	NR	Yes	Yes	Yes	Yes	Performance status. Adjusted for clustering by NHS trust	5
Rich et al, 2011(2) [21]	England	LUCADA and HES	England	2004–2008	Townsend	5	NR	30–100	Yes	Yes	Yes	Yes	Co-morbidity, ethnicity, surgery centre, radiotherapy centre, trial entry. Adjusted for clustering by NHS trust	5
Stevens et al, 2007 [23]	New Zealand	Regional hospital and oncology databases checked against NZ cancer registry	Auckland-Northland region patients managed in secondary care	2004	NZ Deprivation Index	2	NR	<60–80+	Yes	Yes	Yes	Yes	Co-morbidity, private sector care, care discussed at MDM	3
Stevens et al, 2008 [47]	New Zealand	Regional hospital and oncology databases checked against NZ cancer registry	Auckland-Northland region patients managed in secondary care	2004	NZ Deprivation Index	10	NR	<60–80+	Yes	Yes	Yes	Yes	Co-morbidity, private sector care, ethnicity	5

Quality score ranges from 1 (lowest quality) to 6 (highest quality).

a Socioeconomic index (SEI) and household income also measured but individual education level used in analyses as it contained least missing data.

b Odds ratio for 1 unit increase in deprivation score, range unknown.

c Odds ratio for 1 unit increase in deprivation score, range 1–80.

d Quality score 4 where adjusted OR used and 2 where unadjusted rates used.

HES, Hospital Episode Statistics; HES FCE, Hospital Episode Statistics Finished Consultant Episode; IMD, Index of Multiple Deprivation; LUCADA, Lung Cancer Audit; MDM, multi-disciplinary meeting; NCIN/UKACR, National Cancer Information Network/UK Association of Cancer Registries; NR, not reported; OR, odds ratio; SEP, socioeconomic position; UHCS, universal health care system.

**Table 2:** Characteristics of included studies not suitable for meta-analysis (universal health care systems).

Paper	Country of Study	Data Source (s)	Population Included	Years of Diagnosis	Measure of SEP	No of SEP Groups	Treatment Given Within	Age Range	Confounders Controlled For:					Reason for Exclusion	Quality score
									Age	Sex	Stage	Histology	Other		
Battersby et al, 2004 [48]	England	HES and East Anglian Cancer Intelligence Unit	17 PCTs in Norfolk, Suffolk and Cambridgeshire with HES record	1997–2000	IMD (weighted average for PCT)	NR	NR	NR	Yes	Yes	No	Yes	Incidence	Rate correlated against deprivation, by sex	1
Bendzsak et al, 2011 [49]	Canada	Ontario Cancer Registry linked to CIHI hospital data, Insurance data and RPD database	Ontario	2003–2004	Neighbourhood income	5	12 months	20–75+	Yes	Yes	No	No	Univariable analysis	Univariable rate	2
Cartman et al, 2002 [50]	England	Northern and Yorkshire Cancer Registry and Information Service (NYCRIS)	Yorkshire region	1986–1994	NR	NR	NR	<65–75+	Yes	Yes	No	Yes	Univariable analysis	Univariable rate	1
Hui et al, 2005 [51]	Australia	NSW Central Cancer Registry and hospital records	Residents of two area Health Services	1996	SEIFA-IRSD	5	NR	<50–70+	Yes	Yes	Yes	Yes	Univariable analysis	Univariable rate	2
Madelaine et al, 2002 [52]	France	Manche Dept Cancer Registry	Manche	1997–1999	INSEE	4	NR	<54–75+	Yes	Yes	Yes	Yes	Urban/rural	Unemployed used as low SEP group and SEP group 2 used as baseline	2
Pagano et al, 2010 [53]	Italy	Piedmont Cancer Registry of Turin	Turin	2000–2003	Education level	3	12 months	<65–75+	Yes	Yes	Yes	Yes	Marital status	Different comparator – *other* not *no* treatment	2
Patel et al, 2007 [54]	England	Thames Cancer Registry	Southeast England	1994–2003	IMD	5	6 months	0–100	Yes	Yes	Yes	Yes	Cancer network, year of diagnosis	Adjusted rates with no CIs. Possible errors in numbers.	2
Stevens et al, 2009 [55]	New Zealand	Regional hospital and oncology databases checked against NZ cancer Registry listing	Auckland-Northland region patients managed in secondary care	2004	NZ Deprivation Index	10	NR	<60–80+	Yes	Yes	Yes	Yes	Univariable analysis	Univariable OR. Multivariable SEP results not shown	2
Younis et al, 2008 [56]	Canada	Nova Scotia cancer registry and chart review	Nova Scotia	2005	Median household income	2	NR	65–75+	Yes	Yes	Yes	Yes	Co-morbidity, performance status, hospital, surgery type, post-op complications, surgeon, medical oncology, education level, distance to cancer centre, marital status, smoking history	Univariable rate. Multivariable OR only for referral by SEP	2

Quality scores range from 1 (lowest quality) to 6 (highest quality).

CI, confidence interval; HES, Hospital Episode Statistics; IMD, Index of Multiple Deprivation; NR, not reported; NSW, New South Wales; OR, odds ratio; PCT, Primary Care Trust; SEIA-IRSD, Socioeconomic Indexes for Areas - Index of Relative Social Disadvantage; SEP, socioeconomic position; UHCS, universal health care system.

Eighteen papers were from non-UHCSs, all of which were from the USA (Tables 3 and 4). The majority of non-UHCS papers used sub-groups of the National Cancer Institute's Surveillance, Epidemiology and End Results (SEER) database population and, again, some population overlap was found.

**Table 3:** Characteristics of included studies potentially suitable for meta-analysis (non-universal health care systems).

Paper	Country of Study	Data Source (s)	Population Included	Years of Diagnosis	Measure of SEP	No of SEP Groups	Treatment Given Within	Age Range	Confounders Controlled For:					Quality Score
									Age	Sex	Stage	Histology	Other	
Bradley et al, 2008 [57]	USA	Michigan Cancer Registry and Michigan Medicare and Medicaid data	Medicare and Medicare/Medicaid patients in Michigan	1997–2000	Census tract median household income (high v low)	2	6 months	66–80+	Yes	Yes	Yes	Yes	Co-morbidity, insurance type, ethnicity, urban/rural	4
Davidoff et al, 2010 [58]	USA	SEER cancer registry linked to Medicare data	Medicare patients from 16 SEER registries	1997–2002	Census tract median household income	4	90 days	66–85+	Yes	Yes	Yes	Yes	Co-morbidity, performance status, ethnicity, marital status, rural/urban, prior Medicaid, tumour grade	5
Earle et al, 2000 [59]	USA	SEER cancer registry linked to Medicare data	Medicare patients from 11 SEER registries	1991–1993	Census tract median household income(increase in OR per quintile)	5	4 months	65–104	Yes	Yes	Yes	Yes	Co-morbidity, year of diagnosis, ethnicity, rural/urban, teaching hospital, SEER area	5
Esnoala et al, 2008 [60]	USA	South Carolina central cancer Registry linked to inpatient and outpatient surgery files	South Carolina	1996–2002	Income, zip code level (poverty/not living in poverty)	2	NR	<50–80+	Yes	Yes	Yes	Yes	Co-morbidity, year of diagnosis, insurance type, ethnicity, rural/urban, education, marital status, tumour location	4
Greenwald et al, 1998 [61]	USA	SEER cancer registry	3 (Detroit, San Francisco, Seattle) out of 9 SEER registries	1978–1982	Census tract median household income (increase in OR per decile)	10	NR	< = 75	Yes	Yes	Yes	Yes	Performance status, ethnicity	6
Hardy et al, 2009 [62]	USA	SEER cancer registry linked to Medicare data	Medicare patients from 17 SEER registries	1991–2002	% individuals below poverty line at census tract level	4	NR	65–85+	Yes	Yes	Yes	Yes	Co-morbidity, year of diagnosis, ethnicity, marital status, SEER area, other treatment	5
Hayman et al, 2007 [63]	USA	SEER cancer registry linked to Medicare data	Medicare patients from 11 SEER registries	1991–1996	Census tract median household income	5	4 months/2 years	65–85+	Yes	Yes	Yes	Yes	Co-morbidity, year of diagnosis, ethnicity, SEER area, hospitalisation, teaching hospital, distance to nearest RT centre, receipt of chemotherapy	5
Lathan et al, 2008 [64]	USA	SEER cancer registry linked to Medicare data	Medicare patients from 11 SEER registries	1991–1999	Census tract median household income (inc in OR per quintile)	5	NR	65+	Yes	Yes	Yes	Yes	Co-morbidity, ethnicity, SEER registry, urban, non-profit hospital, patient volume, % of black patients in hospital	5
Polednak, 2001 [65]	USA	Connecticut Tumor Registry (SEER) and inpatient hospital discharge database (HDD)	Connecticut	1992–1997	Census tract poverty rate	5	NR	<55–80+	Yes	Yes	Yes	No	Co-morbidity, ethnicity, marital status	4
Smith et al, 1995 [66]	USA	Virginia Cancer Registry and Medicare claims database	Medicare patients from Virginia cancer registry	1985–1989	Census tract: median household income by race and age	Contin-uous^a^	6 months	65–85+	Yes	Yes	Yes	Yes	Co-morbidity, ethnicity, county of residence, distance to oncologist	5

Quality scores range from 1 (lowest quality) to 6 (highest quality).

a Odds ratio for increase per $10,000 income.

CI, confidence interval; non-UHCS, non-universal health care system; NR, not reported; OR, odds ratio; SEER, National Cancer Institute's Surveillance, Epidemiology and End Results database; SEP, socioeconomic position.

**Table 4:** Characteristics of included studies not suitable for meta-analysis (non-universal health care systems).

Paper	Country	Data Source (s)	Population Included	Years of Diagnosis	Measure of SEP	No of SEP Groups	Treatment Given Within	Age Range	Confounders Controlled For:					Reasons for Exclusion	Quality Score
									Age	Sex	Stage	Histology	Other		
Bach et al, 1999 [67]	USA	SEER cancer registry linked to Medicare data	Medicare patients from 10 SEER registries	1985–1993	Median income in zip code of residence (lowest quartile compared to highest 3)	2	NR	65–75+	Yes	Yes	Yes	Yes	Co-morbidity, ethnicity, SEER area	OR of surgery for black v white, univariable rates of surgery used here	2
Earle et al, 2002 [68]	USA	SEER cancer registry linked to Medicare data	Medicare patients from 11 SEER registries	1991–1996	Census tract median household income	5	any time	NR	Yes	Yes	Yes	Yes	Co-morbidity, ethnicity, year of diagnosis, teaching hospital, seen by oncologist, SEER area	SEP non sig in multivariable analysis but only univariable rate shown.	2
Lathan et al, 2006 [69]	USA	SEER cancer registry linked to Medicare data	Medicare patients from 11 SEER registries	1991–1999	Census tract median household income	5	NR	65+	Yes	Yes	Yes	Yes	Co-morbidity, ethnicity, SEER region, teaching hospital, rural/urban	Quality issues	2
Ou et al, 2008 [70]	USA	California Cancer Registry (part of SEER)	California	1989–2003	Composite measure (7 indicators of education, income and occupation)	5	NR	0–89	Yes	Yes	Yes	Yes	Ethnicity, tumour grade, tumour location, histologic grade, marital status	SEP not reported in multivariable analysis. Univariable rate shown.	2
Suga et al, 2010 [71]	USA	California Cancer Registry	Sacramento region in Northern California	1994–2004	Census tract composite variable income, education, employment, poverty, rent, housing value	5	NR	NR	Yes	Yes	Yes	Yes	Ethnicity, residence (urban/rural)	No CIs	2
Tammemagi et al, 2004 [72]	USA	Josephine Ford Cancer Center Tumor Registry	Detroit (receiving care at Henry Ford Health System)	1995–1998	Census tract median household income	Contin-uous^a^	NR	NR	Yes	Yes	Yes	Yes	Co-morbidity, ethnicity, marital status, smoking history, alcohol use, drug use	SEP not reported in multivariable analysis. Univariable OR shown.	2
Wang et al, 2008 [73]	USA	SEER cancer registry linked to Medicare data	Medicare patients 11 SEER registries	1992–2002	% below census tract poverty level	4	4 months	66–85	Yes	Yes	Yes	Yes	Co-morbidity, ethnicity, year of diagnosis, grade, SEER region, census tract education, marital status, teaching hospital, radiation	SEP not reported in multivariable analysis.OR for consultation but not treatment shown.	1
Yang et al, 2010 [74]	USA	Florida Cancer registry linked to inpatient and outpatient medical records	Florida	1998–2002	Census tract poverty level	4	NR	<45–70+	Yes	Yes	Yes	Yes	Univariable analysis only	Univariable rate	2

Quality scores range from 1 (lowest quality) to 6 (highest quality).

a Odds ratio for increase per $10,000 income.

CI, confidence interval; non-UHCS, non-universal health care system; NR, not reported; OR, odds ratio; SEER, National Cancer Institute's Surveillance, Epidemiology and End Results database; SEP, socioeconomic position.

An individual measure of SEP (education level) was used in one study [19]. All other studies used area-level measures of deprivation, income, poverty, or education level.

In terms of quality, the non-UHCS studies that carried out multivariable analyses had better control for confounding than did UHCS studies, as they tended to stratify by stage and histology. However, half of the non-UHCS papers used a Medicare-only population aged over 65, and so were more restrictive in population terms than the UHCS studies.

Twenty-nine papers met the criteria for meta-analysis—19 from UHCSs and 10 from non-UHCSs. However, six studies that examined receipt of treatment in low compared to high SEP presented the results as a single OR and so could not be included in the meta-analyses. Seventeen studies were included in the final meta-analyses and a further six in the sensitivity meta-analyses.

### Surgery

Thirty-one papers (29 study populations) included receipt of surgery as an outcome—18 UHCS papers (15 study populations) and 13 non-UHCS papers (14 study populations) (Tables 5 and 6). Of the papers that reported measures of significance (CIs or p-values), 20 out of 27 (74%) reported that lower SEP was significantly associated with lower likelihood of surgery when comparing the lowest with the highest SEP group, although three of these 20 papers did not find a significant trend across groups.

**Table 5:** Likelihood of receipt of surgery by SEP group (universal health care systems).

Study	No. Receiving Surgery	Cohort No./No. Eligible	Rate	Histology	OR/Rate in Q1 (95% CI)	OR/Rate in Q2 (95% CI)	OR/Rate in Q3 (95% CI)	OR/Rate in Q4 (95% CI)	OR/rate in Q5 (95% CI)	p-Value	Quality Score	Meta-Analysis	Further Information
Bendzsak et al, 2011 [49]	1220	6499	18.77	any	21.1	18.3	19.7	18.8	16.8	0.02	2	N	Univariable rate
Campbell et al, 2002 [35]	85	653	13.02	any	1.00	0.76 (0.28 to 2.09)	0.70 (0.27 to 1.84)	0.88 (0.35 to 2.22)	0.59 (0.23 to 1.53)	0.423	5	Y	P for trend
Hui et al, 2005 [51]	NR	526		any	29	28	20	27	20	0.19	2	N	Univariable rate
Jack et al, 2003 [39]	NR	32818		any					0.98 (0.95 to 1.01)	0.7759	4	N	
Jack et al, 2006 [40]	42	695	6.04	any	1.00	0.82 (0.33 to 2.07)	0.89 (0.35 to 2.25)	0.16 (0.03 to 0.73)	0.75 (0.27 to 2.09)	0.1326	6	Y	Subset of Jack et al (2003) pop, p for trend
Jones et al,2008 [41]	3552	34923	10.17	any					0.99 (0.99 to 1.00)	<0.01	4	N	
Pollock &Vickers, 1998 [44]	2869	38668	7.42	any	1.00	0.83 (0.69 to 1.00)	0.73 (0.61 to 0.88)	0.82 (0.68 to 0.98)	0.58 (0.48 to 0.70)	<0.05	3	Y	Hospital population, p for trend
Raine et al, 2010 [45]	8790	36902	23.82	any	1.63 (1.49 to 1.77)	1.58 (1.46 to 1.72)	1.45 (1.35 to 1.57)	1.34 (1.25 to 1.45)	1.00	<0.001	3	Y	Elective admission population
Raine et al, 2010 [45]	8923	186741	4.78	any	5.5	5.2	4.8	4.4	4.5	NR	2	N	All admissions, univariable rate
Battersby et al, 2004 [48]	387	4092	9.46	NSCLC					−0.10 (−0.55 to 0.40)	NR	1	N	Rate by sex correlated with deprivation score (men), with overall treatment rate
Battersby et al, 2004 [48]				NSCLC					−0.16 (−0.59 to 0.35)	NR	1	N	Rate by sex correlated with deprivation score (women)
Berglund et al, 2010 [19]	626	3369	18.58	NSCLC	1.93 (1.25 to 3.00)		1.33 (0.98 to 1.81)		1.00	NR	6	Y	
Berglund et al, 2010 [19]	534	932	57.30	NSCLC	2.84 (1.40 to 5.79)		1.53 (1.01 to 2.31)		1.00	NR	6	Y(S)	Early stage only - stage IA-IIB
Berglund et al, 2012 [22]	899	1826	49.18	NSCLC	1.00	0.74 (0.51 to 1.06)	0.71 (0.49 to 1.02)	0.73 (0.52 to 1.03)	0.67 (0.48 to 0.95)	0.29	6	Y	Early stage only – stage IA-IIB, p for trend
Cartman et al, 2002 [50]	2401	12570	19.10	NSCLC	19.1				18.6	NR	1	N	Univariable rate
Crawford et al, 2009 [36]	3335	18324	18.20	NSCLC	1.00	0.90 (0.81 to 1.00)		0.82 (0.74 to 0.91)	0.80 (0.72 to 0.89)	<0.05, <0.01, <0.01	4	Y	Individual P values reported
Mahmud et al, 2003 [42]	866	4451	19.46	NSCLC	19.8		18.0		21.0	NR	2	N	Univariable rate
McMahon et al, 2011 [43]	2374	18813	12.62	NSCLC	1.00	0.95 (0.83 to 1.09)	0.95 (0.83 to 1.08)	0.90 (0.79 to 1.03)	0.78 (0.65 to 0.94)	0.018	4	Y	P for trend
									0.96 (0.93 to 0.99)	0.018		N	Paper presents results in 2 different ways
Riaz et al, 2012 [34]	6900	77349	8.92	NSCLC	1.00	0.88 (0.80 to 0.96)	0.91 (0.83 to 0.99)	0.82 (0.76 to 0.89)	0.76 (0.70 to 0.83)	<0.01	4	Y(S)	P for trend
Rich et al, 2011(1) [46]	3427	24175	14.18	NSCLC	1.00	1.13 (0.98 to 1.32)	1.18 (1.02 to 1.37)	1.01 (0.87 to 1.16)	1.11 (0.96 to 1.27)	0.77	5	Y(S)	Subset of Rich et al 2011 (2) pop, p for trend
Rich et al, 2011(2) [21]	4481	34436	13.01	NSCLC	1.00	0.99 (0.88 to 1.11)	1.04 (0.92 to 1.19)	0.98 (0.84 to 1.13)	0.86 (0.71 to 1.04)	0.132	5	Y(S)	P for trend

Some studies reported SEP quintiles but others reported SEP in 2, 3, or 4 categories or as a continuous variable. Details of the number of SEP groups per study are given in Tables 1–4 in the column entitled “No. of SEP groups.” Quality scores range from 1 (lowest quality) to 6 (highest quality). Meta-analysis: Y, included in final meta-analysis; Y(S), included in sensitivity meta-analysis; N, not included in meta-analysis. Q1, high socioeconomic position, Q5, low socioeconomic position.

CI, confidence interval; NR, not reported; OR, odds ratio; pop, population; SEP, socioeconomic position; UHCS, universal health care system.

**Table 6:** Likelihood of receipt of surgery by SEP group (non-universal health care systems).

Study	No. Receiving Surgery	Cohort No./No. Eligible	Rate	Stage(s) Included	Histology	OR/Rate in Q1 (95% CI)	OR/Rate in Q2 (95% CI)	OR/Rate in Q3 (95% CI)	OR/Rate in Q4 (95% CI)	OR/Rate in Q5 (95% CI)	p-Value	Quality Score	Meta-Analysis	Further Information
Bradley et al, 2008 [57]	1336	2626	50.88	I,II,IIIa	NSCLC	1.00				0.80 (0.67 to 0.98)	<0.05	4	Y	
Esnoala et al, 2008 [60]	NR	2791		local	NSCLC	1.00				0.67 (0.51 to 0.88)	0.005	4	Y	
Greenwald et al, 1998 [61]	3053	5157	59.20	I	NSCLC					1.076	<0.0001	6	N	SE = 0.011 (no CIs shown)
Hardy et al, 2009 [62]	11834	19658	60.20	I,II	NSCLC	1.00	0.92 (0.84 to 1.14)		0.78 (0.75 to 1.03)	0.68 (0.60 to 0.77)	>0.05, >0.05, <0.05	5	Y	Individual p values reported corrected OR supplied^a^
Lathan et al, 2008 [64]	4563	9688	47.10	I,II,III	NSCLC					1.06 (1.02 to 1.11)	NR	5	N	Subset of Lathan et al (2006) pop
Ou et al, 2008 [70]	16185	19700	82.16	I	NSCLC	86.9	84.8	81.1	79.6	74.5	<0.001	2	N	
Smith et al, 1995 [66]	801	2813	28.47	local	NSCLC					1.04 (0.90 to 1.19)	>0.001	5	N	
Tammemagi et al, 2004 [72]	NR	1155		I,II	NSCLC					1.19 (1.03 to 1.30)	0.02	2	N	Univariable OR
Bach et al, 1999 [67]	550	860	63.95	I,II	NSCLC	67.5				61.9	NR	2	N	Surgery (blacks)
Bach et al, 1999 [67]	7763	10124	76.68	I,II	NSCLC	78.0				70.7	NR	2	N	Surgery (whites)
Polednak, 2001 [65]	1385	1564	88.55	I,II	NSCLC	1.00	1.27 (0.74 to 2.18)	1.15 (0.65 to 2.03)	1.17 (0.67 to 2.04)	1.78 (1.05 to 3.01)	>0.05, >0.05, >0.05, <0.05	4	Y	Odds of not receiving surgery, individual p values reported
Smith et al, 1995 [66]	57	2396	2.38	distant	NSCLC					1.27 (0.97 to 1.67)	>0.001	5	N	
Suga et al, 2010 [71]	NR	12395			NSCLC					1.17	<0.001	2	N	Surgery after invasive staging, no CIs
Suga et al, 2010 [71]	NR	12395			NSCLC					1.18	<0.001	2	N	Surgery after non-invasive staging, no CIs
Lathan et al, 2006 [69]	NR	14224			NSCLC					1.05 (1.02 to 1.08)	NR	2	N	
Yang et al, 2010 [74]	NR	NR		all	all	24.6	22.2		20.7	18.3	<0.01	2	N	Univariable analysis

Some studies reported SEP quintiles but others reported SEP in 2, 3, or 4 categories or as a continuous variable. Details of the number of SEP groups per study are given in Tables 1–4 in the column entitled “No. of SEP groups.” Quality scores range from 1 (lowest quality) to 6 (highest quality). Meta-analysis: Y, included in final meta-analysis; Y(S), included in sensitivity meta-analysis; N, not included in meta-analysis. Q1, high socioeconomic position; Q5, low socioeconomic position.

a We are grateful to the authors for supplying a corrected OR to allow inclusion of this study in the meta-analysis.

CI, confidence interval; non-UHCS, non-universal health care system; NR, not reported; OR, odds ratio; pop, population; SE, standard error; SEP, socioeconomic position.

**Figure 2:**
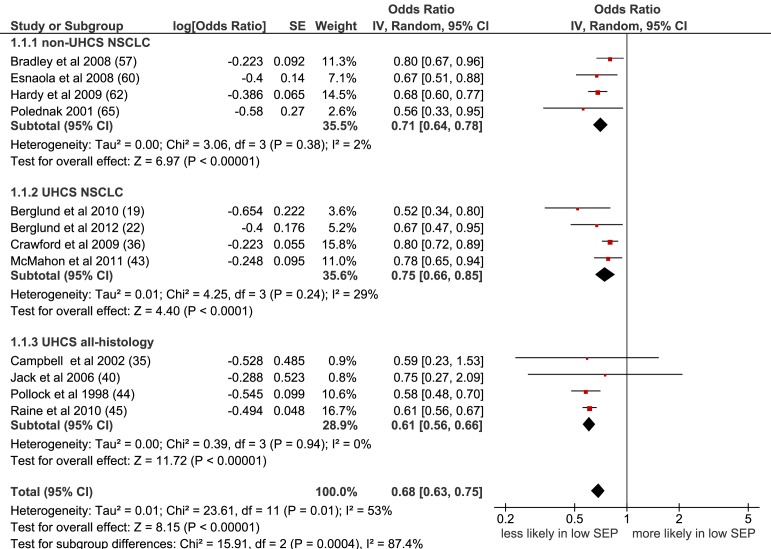
Meta-analysis of odds of receipt of surgery in low versus high SEP. CI, confidence interval; non-UHCS, non-universal health care system; NSCLC, non-small cell lung cancer; OR, odds ratio; SE, standard error; SEP, socioeconomic position; UHCS, universal health care system.

Meta-analysis of all 16 populations that were suitable for inclusion showed a significant negative effect of lower SEP on the likelihood of receiving surgery: OR = 0.72 (95% CI 0.65 to 0.80), p<0.001, I^2^ = 80% (Figure S1). Including only non-overlapping study populations (n = 12) gave a similar result: OR = 0.68 (95% CI 0.63 to 0.75), p<0.001, I^2^ = 53% (Figure 2). Similar results were also seen for the subgroup of eight papers including NSCLC patients only (OR = 0.73 [95% CI 0.68 to 0.80] p<0.001, I^2^ = 24%) (Figure S2) and with further stratification by health care system; NSCLC (UHCS): OR = 0.75 (95% CI 0.66 to 0.85), p<0.001, I^2^ = 29%; NSCLC (non-UHCS, early stage only, co-morbidity included): OR = 0.71 (95% CI 0.64 to 0.78) p<0.001; I^2^ = 2% (Figure 2).

Lower SEP was associated with a lower likelihood of receiving lung cancer surgery, in both types of health care system, and in studies where histology and stage at diagnosis were taken into account.

### Chemotherapy

Twenty-three papers included chemotherapy as an outcome—14 UHCS papers (12 populations) and nine non-UHCS papers (10 populations) (Tables 7 and 8). Of the 21 papers that reported measures of significance, 15 (71%) reported that lower SEP was significantly associated with lower likelihood of receipt of chemotherapy.

**Table 7:** Likelihood of receipt of chemotherapy by SEP group (universal health care systems).

Study	No. Receiving Chemo	Cohort No./No. Eligible	Rate	Histology	OR/Rate in Q1 (95% CI)	OR/Rate in Q2 (95% CI)	OR/Rate in Q3 (95% CI)	OR/Rate in Q4 (95% CI)	OR/Rate in Q5 (95% CI)	p-Value	Quality Score	Meta-Analysis	Further Information
Berglund et al, 2012 [22]	3661	10039	36.47	any	1.00	0.90 (0.77 to 1.06)	0.78 (0.67 to 0.91)	0.77 (0.66 to 0.89)	0.75 (0.65 to 0.87)	<0.01	6	Y	NSCLC stage IIIA-IV & all stage SCLC, p for trend
Campbell et al, 2002 [35]	124	653	18.99	any	1.00	0.58 (0.21 to 1.57)	0.72 (0.29 to 1.78)	0.41 (0.16 to 1.05)	0.39 (0.16 to 0.96)	0.028	5	Y	
Jack et al, 2003 [39]	NR	32818		any					0.96 (0.94 to 0.98)	0.0001	4	N	Subset of Patel et al (2007) pop
Jack et al, 2006 [40]	108	695	15.54	any	1.00	1.04 (0.50 to 2.16)	0.81 (0.38 to 1.70)	0.89 (0.43 to 1.85)	1.04 (0.48 to 2.25)	0.9130	6	Y	Subset of Patel et al (2007) pop, p for trend
Jones et al,2008 [41]	5783	34923	16.56	any					0.99 (0.99 to 0.99)	<0.01	4	N	
Patel et al, 2007 [54]	11217	67312	16.66	any	18.3	15.7	14.5	12.8	12.8	<0.001	2	N	Adjusted rates, no CIs
Rich et al, 2011(1) [46]	14168	59592	23.78	any	1.00	0.97 (0.90 to 1.04)	0.89 (0.83 to 0.96)	0.83 (0.77 to 0.89)	0.85 (0.79 to 0.91)	<0.01	5	Y(S)	
Hui et al, 2005 [51]	NR	526		any	31	34	36	27	26	0.15	2	N	Univariable rate
Berglund et al, 2010 [19]	1285	3369	38.14	NSCLC	1.35 (1.00 to 1.81)		1.25 (1.03 to 1.52)		1.00	NR	6	Y	
Pagano et al, 2010 [53]	430	1231	34.93	NSCLC	1.00		0.98 (0.64 to 1.50)		1.63 (1.08 to 2.44)	NR	2	N	Odds of receiving chemo +/or radio rather than surgery
Younis et al, 2008 [56]	29	108	26.85	NSCLC	4.7 (1.3 to 17.8)				1.0	0.015	2	N	Odds of referral for adjuvant chemo after surgery, stage I, II, III
Cartman et al, 2002 [50]	1349	2448	55.11	SCLC	52.1				56.8	NR	1	N	Univariable rate
Crawford et al, 2009 [36]	3619	5510	65.68	SCLC	1.00	1.10 (0.94 to 1.30)		0.91 (0.78 to 1.08)	0.94 (0.80 to 1.11)	>0.05	4	Y	Individual p-values, all reported as >0.05
Mahmud et al, 2003 [42]	425	1002	42.42	SCLC	37.8		40.5		50.2	NR	2	N	Univariable rate

Some studies reported SEP quintiles but others reported SEP in 2, 3, or 4 categories or as a continuous variable. Details of the number of SEP groups per study are given in Tables 1–4 in the column entitled “No. of SEP groups.” Quality scores range from 1 (lowest quality) to 6 (highest quality). Meta-analysis: Y, included in final meta-analysis; Y(S), included in sensitivity meta-analysis; N, not included in meta-analysis. Q1, high socioeconomic position; Q5, low socioeconomic position.

CI, confidence interval; NR, not reported; OR, odds ratio; pop, population; SEP, socioeconomic position; UHCS, universal health care system.

**Table 8:** Likelihood of receipt of chemotherapy by SEP group (non-universal health care systems).

Study	No. Receiving Chemo	Cohort No./No. Eligible	Rate	Stage	Histology	OR/Rate in Q1 (95% CI)	OR/Rate in Q2 (95% CI)	OR/Rate in Q3 (95% CI)	OR/Rate in Q4 (95% CI)	OR/Rate in Q5 (95% CI)	p-Value	Quality Score	Meta-Analysis	Further Information
Bradley et al, 2008 [57]	643	2348	27.39	I,II, IIIa	NSCLC	1.00				1.09 (0.87 to 1.37)	>0.05	4	Y	
Hardy et al, 2009 [62]	2951	19658	15.01	I, II	NSCLC	1.00	0.91 (0.81 to 1.02)		0.96 (0.85 to 1.09)	0.85 (0.74 to 0.98)	>0.05, >0.05, <0.05	5	Y	Individual p-values reported
Ou et al, 2008 [70]	1175	19700	5.96	I	NSCLC	5.3	5.7	5.3	6.9	7.4	0.001	2	N	Univariable analysis
Davidoff et al, 2010 [58]	5499	21285	25.84	IIIB, IV	NSCLC	1.43 (1.28 to 1.60)	1.17 (1.05 to 1.30)		1.11 (1.00 to 1.22)	1.00	<0.01, <0.01, <0.05	5	Y	Individual p-values reported
Earle et al, 2000 [59]	1356	6308	21.50	IV	NSCLC					1.07 (1.02 to 1.12)	0.0077	5	N	Subset of Earle (2002)
Earle et al, 2002 [68]	8813	12015	73.35	IV	NSCLC	41	41	36	31	27	>0.05	2	N	Univariable analysis only. SEP was included in multivariable analysis but non-sig (figs not reported)
Hardy et al, 2009 [62]	26417	51243	51.55	III, IV	NSCLC	1.00	0.87 (0.78 to 0.96)		0.76 (0.63 to 0.90)	0.60 (0.45 to 0.79)	<0.05, <0.05, <0.05	5	Y(S)	Individual p-values reported
Tammemagi et al, 2004 [72]	NR	1155		III,IV	NSCLC					1.09 (1.01 to 1.18)	0.03	2	N	Univariable OR
Davidoff et al, 2010 [58]	749	1946	38.49	IIIB, IV	NSCLC	0.86(0.69 to 1.08)	0.96 (0.77 to 1.19)		0.99 (0.81 to 1.22)	1.00	NR	5	N	Odds of single agent compared to two-agent chemo.
Wang et al, 2008 [73]	1521	3196	47.59	II, IIIa	NSCLC	1.00	1.08 (0.97 to 1.21)		1.08 (0.97 to 1.21)	0.97 (0.85 to 1.10)	NR	1	N	Odds of receiving oncology consultation.
Yang et al, 2010 [74]	NR	NR		All	any	32.2	30.7		29.9	30.1	<0.01	2	N	Univariable analysis

Some studies reported SEP quintiles but others reported SEP in 2, 3, or 4 categories or as a continuous variable. Details of the number of SEP groups per study are given in Tables 1–4 in the column entitled “No. of SEP groups.” Quality scores range from 1 (lowest quality) to 6 (highest quality). Meta-analysis: Y, included in final meta-analysis; Y(S), included in sensitivity meta-analysis; N, not included in meta-analysis. Q1, high socioeconomic position; Q5, low socioeconomic position.

CI, confidence interval; non-UHCS, non-universal health care system; NR, not reported; OR, odds ratio; pop, population; SEP, socioeconomic position.

**Figure 3:**
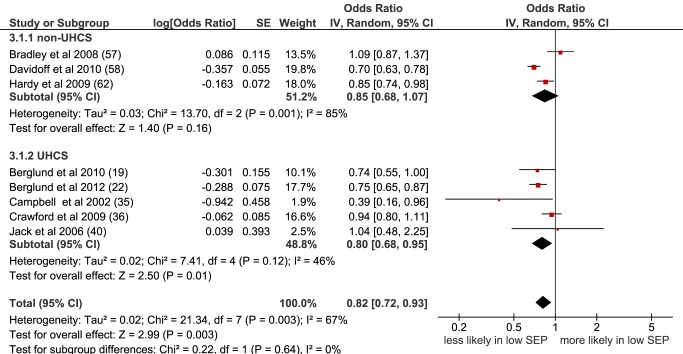
Meta-analysis of odds of receipt of chemotherapy in low versus high SEP. CI, confidence interval; non-UHCS, non-universal health care system; OR, odds ratio; SE, standard error; SEP, socioeconomic position; UHCS, universal health care system.

Meta-analysis of the ten populations that were suitable for inclusion found a significant negative effect of lower SEP on the likelihood of receiving chemotherapy: OR = 0.81 (95% CI 0.73 to 0.89), p<0.001, I^2^ = 68% (Figure S3). Similarly, in a meta-analysis of the eight papers containing non-overlapping populations that were selected for inclusion, the odds of receiving chemotherapy were significantly lower for those with low SEP compared to those with high SEP (OR = 0.82 [95% CI 0.72 to 0.93], p = 0.003, I^2^ = 67%), overall. A similar pattern was found in UHCS (OR = 0.80 [95% CI 0.68 to 0.95], p = 0.01, I^2^ = 46%); and in non-UHCS settings (OR = 0.85 [95% CI 0.68 to 1.07], p = 0.16, I^2^ = 85%), although this did not reach significance (Figure 3).

### Radiotherapy

Eighteen papers (18 populations) examined receipt of radiotherapy for lung cancer—12 in UHCS settings (11 populations) and six in non-UHCS settings (seven populations) (Tables 9 and 10). Only one UHCS study found an association between SEP and receipt of radiotherapy. The non-UHCS studies had very heterogeneous outcomes.

**Table 9:** Likelihood of receipt of radiotherapy by SEP group (universal health care systems).

Study	No. Receiving Radio	Cohort No./No. Eligible	Rate	Histology	OR/Rate in Q1 (95% CI)	OR/Rate in Q2 (95% CI)	OR/Rate in Q3 (95% CI)	OR/Rate in Q4 (95% CI)	OR/Rate in Q5 (95% CI)	p-Value	Quality Score	Meta-Analysis	Further Information
Berglund et al, 2012 [22]	1054	2771	38.04	any	1.00	1.16 (0.88 to 1.54)	1.17 (0.90 to 1.53)	1.18 (0.91 to 1.53)	0.99 (0.77 to 1.29)	0.67	6	Y	Stage III only, p for trend
Campbell et al, 2002 [35]	412	653	63.09	any	1,00	2.08 (1.11 to 3.91)	2.27 (1.24 to 4.16)	1.47 (0.83 to 2.60)	1.86 (1.05 to 3.28)	0.378	5	Y	P for trend
Jack et al, 2003 [39]	NR	32818		any					1.00 (0.99 to 1.02)	0.2048	4	N	
Jack et al, 2006 [40]	338	695	48.63	any	1.00	1.24 (0.76 to 2.02)	0.76 (0.46 to 1.26)	0.98 (0.60 to 1.59)	0.68 (0.41 to 1.14)	0.0978	6	Y	Subset of Jack et al (2003) pop, p for trend
Jones et al,2008 [41]	13857	34923	39.68	any					0.99 (0.99 to 1.00)	<0.01	4	N	
Rich et al, 2011(1) [46]	12079	59592	20.27	any	1.00	1.08 (1.01 to 1.16)	1.12 (1.04 to 1.20)	1.12 (1.04 to 1.20)	1.02 (0.95 to 1.09)	0.80	5	Y(S)	P for trend
Hui et al, 2005 [51]	NR	526		any	52	62	51	55	55	0.84	2	N	Univariable rate
Stevens et al, 2009 [55]	222	555	40.00	any	1.0	0.8 (0.4 to 1.5)	0.6 (0.3 to 1.2)	0.9 (0.5 to 1.6)	0.7 (0.4 to 1.3)	>0.05	2	N	Hosp pop, univariable OR
Berglund et al, 2010 [19]	863	3369	25.62	NSCLC	0.91 (0.67 to 1.22)		1.12 (0.93 to 1.36)		1.00	NR	6	Y	
Erridge et al, 2002 [37]	824	3177	25.94	NSCLC/unknown	1.00	0.94 (0.70 to 1.26)	1.04 (0.79 to 1.38)	1.33 (1.01 to 1.75)	1.13 (0.84 to 1.51)	0.10	6	Y	
Mahmud et al, 2003 [42]	1265	4451	28.42	NSCLC	26.1		29.0		29.9	NR	2	N	Univariable rate
Cartman et al, 2002 [50]	693	2448	28.31	SCLC	37.1				39.5	NR	1	N	Univariable rate

Some studies reported SEP quintiles but others reported SEP in 2, 3, or 4 categories or as a continuous variable. Details of the number of SEP groups per study are given in Tables 1–4 in the column entitled “No. of SEP groups.” Quality scores range from 1 (lowest quality) to 6 (highest quality). Meta-analysis: Y, included in final meta-analysis; Y(S), included in sensitivity meta-analysis; N, not included in meta-analysis. Q1, high socioeconomic position; Q5, low socioeconomic position.

CI, confidence interval; NR, not reported; OR, odds ratio; pop, population; SEP, socioeconomic position; UHCS, universal health care system.

**Table 10:** Likelihood of receipt of radiotherapy by SEP group (non-universal health care systems).

Study	No. Receiving Radio	Cohort No./No. Eligible	Rate	Stage	Histology	OR/rate in Q1 (95% CI)	OR/rate in Q2 (95% CI)	OR/rate in Q3 (95% CI)	OR/rate in Q4 (95% CI)	OR/rate in Q5 (95% CI)	P value	Quality Score	Meta-analysis	Further information
Bradley et al, 2008 [57]	950	2348	40.46	I,II,IIIa	NSCLC	1.00				0.97 (0.79 to 1.19)	>0.05	4	Y	
Ou et al, 2008 [70]	2779	19700	14.11	I	NSCLC	11.7	12.6	14.7	16.5	16.6	<0.001	2	N	Univariable analysis
Smith et al, 1995 [66]	1323	2813	47.03	local	NSCLC					0.95 (0.83 to 1.09)	>0.001	5	N	
Hardy et al, 2009 [62]	43519	51243	84.93	III,IV	NSCLC	1.00	1.01 (0.96 to 1.07)		0.93 (0.88 to 0.99)	0.88 (0.82 to 0.93)	0.05, <0.05, <0.05	5	Y	Individual p-values reported
Hayman et al, 2007 [63]	6436	11084	58.07	IV	NSCLC	1.48 (1.17 to 1.87)	1.50 (1.17 to 1.91)	1.32 (1.01 to 1.72)	1.25 (0.93 to 1.69)	1.00	<0.001	5	Y(S)	
Smith et al, 1995 [66]	1438	2396	60.02	distant	NSCLC					1.00 (0.90 to 1.12)	>0.001	5	N	
Yang et al, 2010 [74]	NR	NR		??	any	32.0	32.1		31.4	33.1	0.02	2	N	Univariable analysis

Some studies reported SEP quintiles but others reported SEP in 2, 3, or 4 categories or as a continuous variable. Details of the number of SEP groups per study are given in Tables 1–4 in the column entitled “No. of SEP groups.” Quality scores range from 1 (lowest quality) to 6 (highest quality). Meta-analysis: Y, included in final meta-analysis; Y(S), included in sensitivity meta-analysis; N, not included in meta-analysis. Q1, high socioeconomic position; Q5, low socioeconomic position.

CI, confidence interval; non-UHCS, non-universal health care system; NR, not reported; OR, odds ratio; pop, population; SE, standard error; SEP, socioeconomic position.

**Figure 4:**
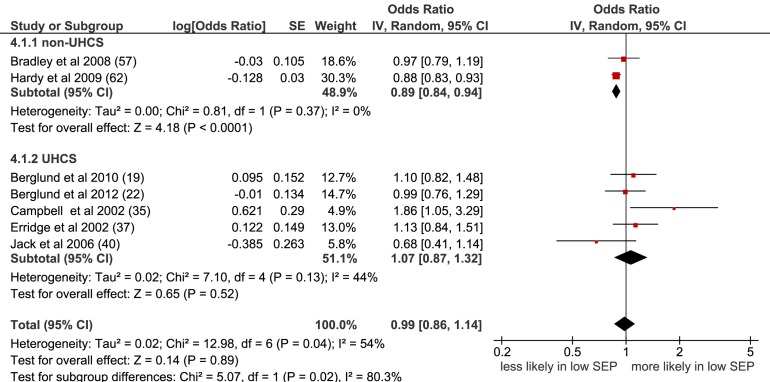
Meta-analysis of odds of receipt of radiotherapy in low versus high SEP. CI, confidence interval; non-UHCS, non-universal health care system; OR, odds ratio; SE, standard error; SEP, socioeconomic position; UHCS, universal health care system.

Overall, no association between SEP and receipt of radiotherapy was seen in the meta-analysis of the seven studies with non-overlapping populations selected for inclusion (OR = 0.99 [95% CI 0.86 to 1.14], p = 0.89, I^2^ = 54%) (Figure 4), or when all nine studies were included (OR = 0.95 [95% CI 0.85 to 1.06], p = 0.40, I^2^ = 71%) (Figure S4). A significant association was seen for non-UHCS studies but only two studies were included here, each looking at different stage patients. 

### Treatment Type not Specified

**Figure 5:**
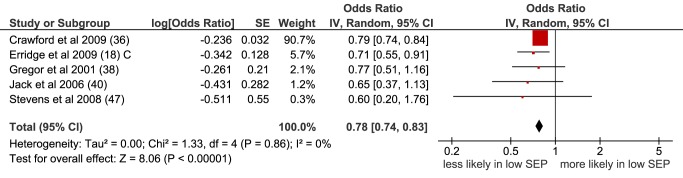
Meta-analysis of odds of receipt of unspecified treatment in low versus high SEP. CI, confidence interval; OR, odds ratio; SE, standard error; SEP, socioeconomic position.

Seven papers (eight study populations) examined receipt of unspecified treatment, and three papers considered receipt of unspecified curative treatment in three populations (Tables 11–13). In the meta-analysis of five non-overlapping studies, low SEP was associated with a lower likelihood of receiving unspecified treatment (OR = 0.78 [95% CI 0.74 to 0.83], p<0.001, I^2^ = 0) (Figure 5). This was also seen when studies with overlapping populations were included (OR = 0.80 [95% CI 0.77 to 0.84], p<0.001, I^2^ = 17%) (Figure S5).

**Table 11:** Likelihood of receipt of any type of unspecified treatment by SEP group (universal health care systems).

Study	No. Receiving Treatment	Cohort No./No. Eligible	Rate	Histology	OR/Rate in Q1 (95% CI)	OR/Rate in Q2 (95% CI)	OR/Rate in Q3 (95% CI)	OR/Rate in Q4 (95% CI)	OR/Rate in Q5 (95% CI)	p-Value	Quality Score	Meta-Analysis	Further Information
Crawford et al, 2009 [36]	19667	34923	56.32	any	1.00	0.91 (0.86 to 0.97)		0.82(0.77 to 0.88)	0.79 (0.74 to 0.84)	<0.01	4	Y	Individual p-values, all reported as <0.01
Erridge et al, 2009 [18]	2186	3833	57.03	any	1.3 (1.1 to 1.5)				1.00	<0.05	4	Y(S)	Scottish population
Erridge et al, 2009 [18]	1372	2073	66.18	any	1.3 (1.1 to 1.7)				1.00	<0.05	4	Y(S)	Canadian population
Jack et al, 2003 [39]	NR	32818		any					0.98 (0.96 to 0.99)	0.0091	4	N	
Jack et al, 2006 [40]	414	695	59.57	any	1.00	0.91 (0.53 to 1.55)	0.69 (0.40 to 1.19)	0.57 (0.34 to 0.97)	0.65 (0.37 to 1.13)	0.03	6	Y	Subset of Jack et al (2003) population, p for trend
Stevens et al, 2007 [23]	285	565	50.44	any	1.0				0.9 (0.6 to 1.5)	0.773	3	Y(S)	Hospital population
Mahmud et al, 2003 [42]	2678	4451	60.17	NSCLC	1.0		0.9 (0.8 to 1.1)		1.0 (0.8 to 1.2)	0.39, 0.958	4	Y(S)	Odds of NOT receiving treatment—individual p-values reported
Mahmud et al, 2003 [42]	694	1002	69.26	SCLC	1.0		1.0 (0.6 to 1.5)		0.8 (0.5 to 1.3)	0.888, 0.358	4	Y(S)	Odds of NOT receiving treatment—individual p-values reported

Some studies reported SEP quintiles but others reported SEP in 2, 3, or 4 categories or as a continuous variable. Details of the number of SEP groups per study are given in Tables 1–4 in the column entitled “No. of SEP groups.” Quality scores range from 1 (lowest quality) to 6 (highest quality). Meta-analysis: Y, included in final meta-analysis; Y(S), included in sensitivity meta-analysis; N, not included in meta-analysis. Q1, high socioeconomic position; Q5, low socioeconomic position.

CI, confidence interval; NR, not reported; OR, odds ratio; SEP, socioeconomic position; UHCS, universal health care system.

**Table 12:** Likelihood of receipt of any type of unspecified treatment by SEP group (non-universal health care systems).

Study	No. Receiving Treatment	Cohort No./No. Eligible	Rate	Histology	OR/Rate in Q1 (95% CI)	OR/Rate in Q2 (95% CI)	OR/Rate in Q3 (95% CI)	OR/Rate in Q4 (95% CI)	OR/Rate in Q5 (95% CI)	p-Value	Quality Score	Meta-Analysis	Further Information
Ou et al, 2008 [70]	18216	19700	92.47	NSCLC	94.7	94.1	92.2	91.9	87.2	<0.001	2	N	Stage I. Univariable analysis
Smith et al, 1995 [66]	1697	2396	70.83	NSCLC					1.00 (0.91 to 1.11)	>0.001	5	N	Distant stage
Smith et al, 1995 [66]	2343	2813	83.29	NSCLC					1.00 (0.88 to 1.13)	>0.001	5	N	Local stage

Some studies reported SEP quintiles but others reported SEP in 2, 3, or 4 categories or as a continuous variable. Details of the number of SEP groups per study are given in Tables 1–4 in the column entitled “No. of SEP groups.” Quality scores range from 1 (lowest quality) to 6 (highest quality). Meta-analysis: Y, included in final meta-analysis; Y(S), included in sensitivity meta-analysis; N, not included in meta-analysis. Q1, high socioeconomic position; Q5, low socioeconomic position.

CI, confidence interval; non-UHCS, non-universal health care system; NR, not reported; OR, odds ratio; pop, population; SE, standard error; SEP, socioeconomic position.

**Table 13:** Likelihood of receipt of any type of unspecified curative treatment by SEP group (universal health care systems).

Study	No. Receiving Treatment	Cohort No. / No. Eligible	Rate/ Eligible Rate	Histology	OR/Rate in Q1 (95% CI)	OR/Rate in Q2 (95% CI)	OR/Rate in Q3 (95% CI)	OR/Rate in Q4 (95% CI)	OR/Rate in Q5 (95% CI)	p-Value	Quality Score	Meta-Analysis	Further Information
Erridge et al, 2009 [18]	548	3833	14.30	any	1.1(0.9 to 1.4)				1.00	>0.05	4	Y (S)	Scottish pop – subset of Gregor et al (2001) pop
Erridge et al, 2009 [18]	546	2073	26.34	any	1.4(1.1 to 1.8)				1.00	<0.05	4	Y	Canadian pop
Gregor et al, 2001 [38]	627	3855/1423	16.26/44.06	any	1.00	1.14 (0.72 to 1.80)	1.07 (0.69 to 1.66)	0.95 (0.62 to 1.47)	0.77 (0.51 to 1.16)	0.25	6	Y	Eligible = early stage
Stevens et al, 2008 [47]	109	565	19.29	any	1.0	3.1 (1.0 to 9.7)	1.4 (0.4 to 4.4)	1.1 (0.4 to 0.3)	0.6 (0.2 to 1.8)	0.05, 0.60, 0.86, 0.40	5	Y	Hospital pop - subset of Stevens et al (2007) pop, individual p-values reported

Some studies reported SEP quintiles but others reported SEP in 2, 3, or 4 categories or as a continuous variable. Details of the number of SEP groups per study are given in Tables 1–4 in the column entitled “No. of SEP groups.” Quality scores range from 1 (lowest quality) to 6 (highest quality). Meta-analysis: Y, included in final meta-analysis; Y(S), included in sensitivity meta-analysis; N, not included in meta-analysis. Q1, high socioeconomic position, Q5, low socioeconomic position.

CI, confidence interval; NR, not reported; OR, odds ratio; pop, population; SEP, socioeconomic position; UHCS, universal health care system.

When the surgery, chemotherapy, and radiotherapy papers included in the separate treatment meta-analyses in this systematic review were analysed together to produce an overall summary effect meta-analysis OR, a similar result was seen, with low SEP associated with a lower likelihood of receiving any type of treatment. This was found when including only studies with non-overlapping populations (OR = 0.79 [95% CI 0.73 to 0.86], p<0.001, I^2^ = 77%) (Figure S6) and when including all eligible studies (OR = 0.80 [95% CI 0.75 to 0.86], p<0.001, I^2^ = 82%) (Figure S7).

## Discussion

### Principal Findings

To our knowledge, this is the first systematic review and meta-analysis examining socioeconomic inequalities in receipt of lung cancer treatment. It shows an association between low SEP and reduced likelihood of receipt of any type of treatment, surgery, and chemotherapy. The results were generally consistent across different health care systems.

### Interpretation of Results

Surgery is suitable only for patients with early-stage NSCLC, and it has been suggested that patients with cancer in a lower SEP are more likely to present later and with later-stage disease [20]. This may help explain why socioeconomic inequalities in receipt of surgery are observed in some studies. However, presentation with later-stage cancer in lower SEP patients has not been consistently observed [19]. In this review, when receipt of treatment was examined in studies of early-stage patients only (from non-UHCS studies), low SEP remained associated with reduced likelihood of surgery. Thus, the association between SEP and receipt of surgery appears to be independent of stage. Similar results were seen for NSCLC studies in both health care systems.

Receipt of treatment may also be influenced by clinical suitability for treatment, and socioeconomic differences in the number of co-morbidities present may explain socioeconomic inequalities in treatment. In the three UHCS studies that took co-morbidity into account, SEP was not associated with receipt of surgery [21],[22] or of any treatment [23] when the trend across SEP groups was examined, suggesting that co-morbidity may be a potential mediator of socioeconomic inequalities in treatment in UHCSs. However, most of the non-UHCS studies did include co-morbidity as a confounder, and socioeconomic inequalities in treatment were still observed, suggesting that there may be differences between health care systems here.

### Strengths and Weaknesses of the Review and of the Available Evidence

This is one of the first equity reviews published [24],[25], the first systematic review of the literature on intervention-generated inequalities in lung cancer treatment to our knowledge, and the first cancer equity review to include a meta-analysis. Extensive searches were carried out to identify studies. However, it is possible that not all relevant studies were obtained.

The included studies reported observational data only. The suitability of meta-analysis for observational studies has been questioned, as it may produce precise but spurious results [26]. Examining the possible sources of heterogeneity by conducting sensitivity analyses across different sub-groups may be less prone to bias than calculating an overall summary effect [26]. Here, although an overall summary effect OR was calculated, heterogeneity was taken into account. Separate analyses by type of treatment were carried out, with further stratification by stage and histology. Universal and non-UHCSs were examined separately and random effects rather than fixed effects meta-analyses were conducted. These precautions did not change the overall pattern of results seen.

Significant heterogeneity remained in some cases, which could be considered a limitation, although this is not surprising because of the characteristics of the studies included. For studies examining receipt of chemotherapy and radiotherapy it was generally not possible to differentiate between curative and palliative treatment and, if patterns of care differ for these by SEP, this might explain the high degree of heterogeneity seen. However, although there is some suggestion that heterogeneity can be considered high at >50% [17], when confidence intervals were calculated these were wide, so it was difficult to be confident about the degree of heterogeneity present [27].

Results for receipt of radiotherapy differed in the non-UHCS sub-group compared to overall but, as only two studies were included in this sub-group, it is difficult to be sure that different patterns of receipt of radiotherapy by SEP are due to differences in health care system.

Many of the non-UHCS studies used overlapping population sub-groups from the SEER database. There was also population overlap between some UHCS datasets. We attempted to include only substantially non-overlapping datasets within the final meta-analyses to ensure independence of results. A judgement had to be made as to which was the best-quality and most appropriate paper to include, but sensitivity analyses using different inclusion combinations (Figure S8) did not change the overall findings, nor did including all suitable studies regardless of population overlap (Figures S1, S3, S4, S5, S7).

Included papers contained data for patients diagnosed between 1978 and 2008. As treatment guidance has changed over time, older studies may be less applicable to current clinical practice. However, the majority of included studies were published within the last five years, and sensitivity analyses excluding studies published prior to 2000 did not change the overall findings.

Various measures of SEP were used, and these were categorised differently—an acknowledged problem in equity reviews [28]. All but one study measured SEP at the area level. This is a further limitation, as area-based measures of SEP are unlikely to be accurate markers of individual-level circumstances and access to resources [29]. Area-based measures of SEP can be calculated using address, making them easy to add to disease registers, such as those used in many of the studies synthesised here. However, the reliance on area-based markers of SEP may underestimate the strength of the true association between SEP and receipt of treatment.

Not all studies reported details of stage and histology—both of which influence treatment type—and very few UHCS studies took co-morbidity into account. Thus, the ORs used in the meta-analyses were not consistently adjusted for the same covariates. However, we attempted to take these factors into account in the quality scores and by conducting subgroup sensitivity analyses. Examining only high-quality studies did not alter our findings nor did sensitivity analyses, although consequent reduction in numbers did result in loss of significance in some analyses, potentially due to lack of power to detect differences.

In order to conduct meta-analysis it is necessary to compare the odds of treatment in the lowest-SEP group with the odds in the highest, which simplifies what may be a complex relationship across SEP groups. However, studies that reported a change in odds ratios across the SEP categories, and thus explored trends in receipt of treatment, generally supported the overall findings of the review.

A number of existing tools suitable for assessing cohort study quality were considered [15],[16]. However, none of these tools was entirely appropriate for the type of studies included and, as has been done in previous reviews [13],[30], we devised a unique tool, adapting and utilising aspects of other available tools. This approach has the benefit of producing a quality tool that is highly specific for the type of studies examined.

As with any systematic review, we are unable to exclude the possibility of publication bias. Studies reporting null findings are less likely to be published or, if they are published, not to report numerical outcomes [17]. A funnel plot to assess potential publication bias did not show obvious bias (Figure S9). However, a number of papers recovered in the search included SEP in the description of the study population but did not report receipt of treatment by SEP [31]–[34]. Study authors were contacted and asked to provide further information, but only one supplied the requested data [34]. It is likely that SEP was not significantly associated with receipt of treatment in the other studies, but this was not always clearly reported. However, publication bias is thought to be less important than other sources of bias, such as confounding, in meta-analyses of observational studies [26].

### Implications for Policy and Practice

Socioeconomic inequalities in receipt of treatment may exacerbate socioeconomic inequalities in incidence of lung cancer, which is strongly associated with higher smoking rates in more deprived populations, so may further contribute to the poorer outcomes in lower SEP groups.

Socioeconomic inequalities in treatment may be due to differences in access to care. Within a non-UHCS it might be expected that socioeconomic differences in receipt of treatment would be observed due to income-related differences in health insurance status. Patients with lung cancer in the USA who do not have insurance have been shown to have more limited access to care [13]. However, as socioeconomic inequalities in receipt of lung cancer treatment were also observed in UHCSs that do not depend on ability to pay and in non-UHCS studies where insurance type was taken into account, this would suggest that other system factors may be contributing to this inequality. The extent to which receipt of treatment is influenced by factors such as patient choice is not known.

Variability at the patient, tumour, system, and individual clinician levels needs to be investigated before clear recommendations for changes to policy and practice can be made.

### Future Research

This review has demonstrated a clear association between lower SEP and reduced likelihood of receiving surgery, chemotherapy, and any type of unspecified treatment for lung cancer. The reasons for these inequalities need to be more thoroughly investigated. Better-quality UHCS studies, including statistical control for co-morbidity and stratification by stage and histology—so that only those patients eligible for a particular treatment are included in the population-denominator—are required. It would also be useful to be able to distinguish between curative and palliative intent of treatment. In non-UHCS, studies in younger populations, examining a range of insurance providers, are required.

Further investigation into the system and patient factors that might contribute to socioeconomic inequalities in receipt of lung cancer care is necessary, to help develop interventions that ensure equitable receipt of appropriate treatment. This should include a quantitative exploration of inequalities at each stage of the care pathway as well as qualitative work exploring reasons for inequality. Inequalities in receipt of treatment may contribute to inequalities in cancer survival and so cohort survival analyses are warranted in order to investigate intervention-generated inequalities in lung cancer outcomes.

## Supporting Information

Figure S1 **Meta-analysis of odds of receipt of surgery in low versus high SEP (overlapping populations).** CI, confidence interval; non-UHCS, non-universal health care system; OR, odds ratio; SE, standard error; SEP, socioeconomic position; UHCS, universal health care system.
(TIF)

Figure S2 **Meta-analysis of odds of receipt of surgery for NSCLC in low versus high SEP (non-overlapping populations).** CI, confidence interval; OR, odds ratio; SE, standard error; SEP, socioeconomic position.
(TIF)

Figure S3 **Meta-analysis of odds of receipt of chemotherapy in low versus high SEP (overlapping populations).** CI, confidence interval; non-UHCS, non-universal health care system; OR, odds ratio; SE, standard error; SEP, socioeconomic position; UHCS, universal health care system.
(TIF)

Figure S4 **Meta-analysis of odds of receipt of radiotherapy in low versus high SEP (overlapping populations).** CI = confidence interval, non-UHCS = non-universal health care system, OR = odds ratio, SE = standard error, SEP = socioeconomic position UHCS = universal health care system.
(TIF)

Figure S5 **Sensitivity meta-analysis of odds of receipt unspecified treatment in low versus high SEP (overlapping populations).** CI, confidence interval; OR, odds ratio; SE, standard error; SEP, socioeconomic position.
(TIF)

Figure S6 **Meta-analysis of odds of receipt of any type of treatment in low versus high SEP.** CI, confidence interval; non-UHCS, non-universal health care system; OR, odds ratio; SE, standard error; SEP, socioeconomic position; UHCS, universal health care system.
(TIF)

Figure S7 **Meta-analysis of odds of receipt of any type of treatment in low versus high SEP (overlapping populations).** CI, confidence interval; non-UHCS, non-universal health care system; OR, odds ratio; SE, standard error; SEP, socioeconomic position; UHCS, universal health care system.
(TIF)

Figure S8 **Meta-analysis of odds of receipt of surgery in low versus high SEP (partially-overlapping populations).** CI, confidence interval; non-UHCS, non-universal health care system; OR, odds ratio; SE, standard error; SEP, socioeconomic position; UHCS, universal health care system.
(TIF)

Figure S9 **Funnel plot to assess publication bias.** CI, confidence interval; non-UHCS, non-universal health care system; NSCLC, non-small cell lung cancer; UHCS, universal health care system.
(TIF)

Table S1 **Full search strategies (MEDLINE and EMBASE).**
(DOC)

Text S1 **PRISMA checklist.**
(DOC)

Text S2 **Protocol.**
(DOC)

Text S3 **Quality score checklist.**
(DOC)

## Abbreviations

CI: confidence interval
NSCLC: non-small cell lung cancer
OR: odds ratio
SCLC: small cell lung cancer
SEER: National Cancer Institute's Surveillance, Epidemiology and End Results
SEP: socioeconomic position
UHCS: universal health care system
