# Supplementary figures and images for: Socioeconomic Inequalities in Lung Cancer Treatment: Systematic Review and Meta-Analysis
Source: PLoS Med. 2013 Feb 5;10(2):e1001376. doi: 10.1371/journal.pmed.1001376 (PMC3564770; doi:10.1371/journal.pmed.1001376)

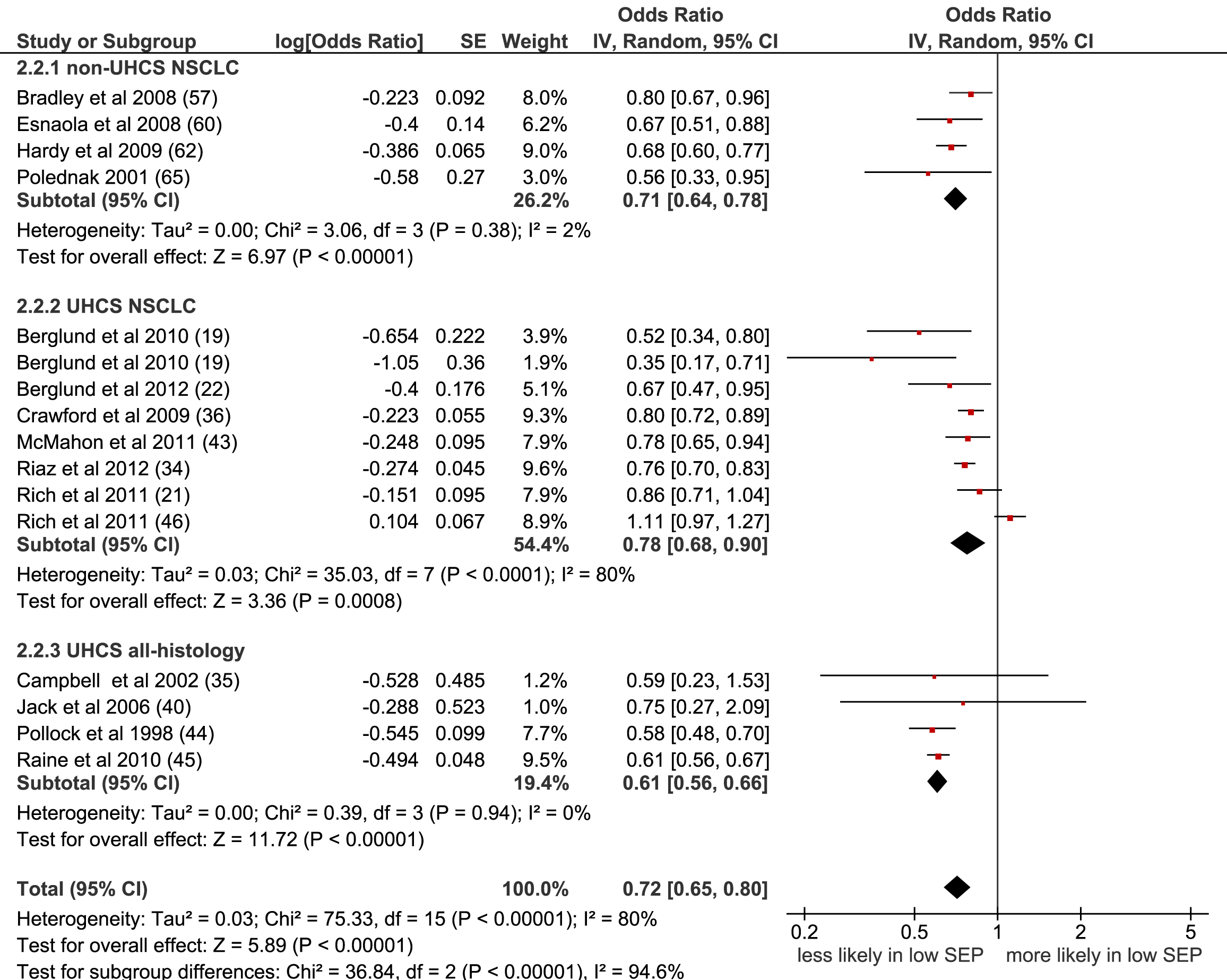

Supplement: Figure S1 — Meta-analysis of odds of receipt of surgery in low versus high SEP (overlapping populations). CI, confidence interval; non-UHCS, non-universal health care system; OR, odds ratio; SE, standard error; SEP, socioeconomic position; UHCS, universal health care system. (TIF) [file pmed.1001376.s001.tif]

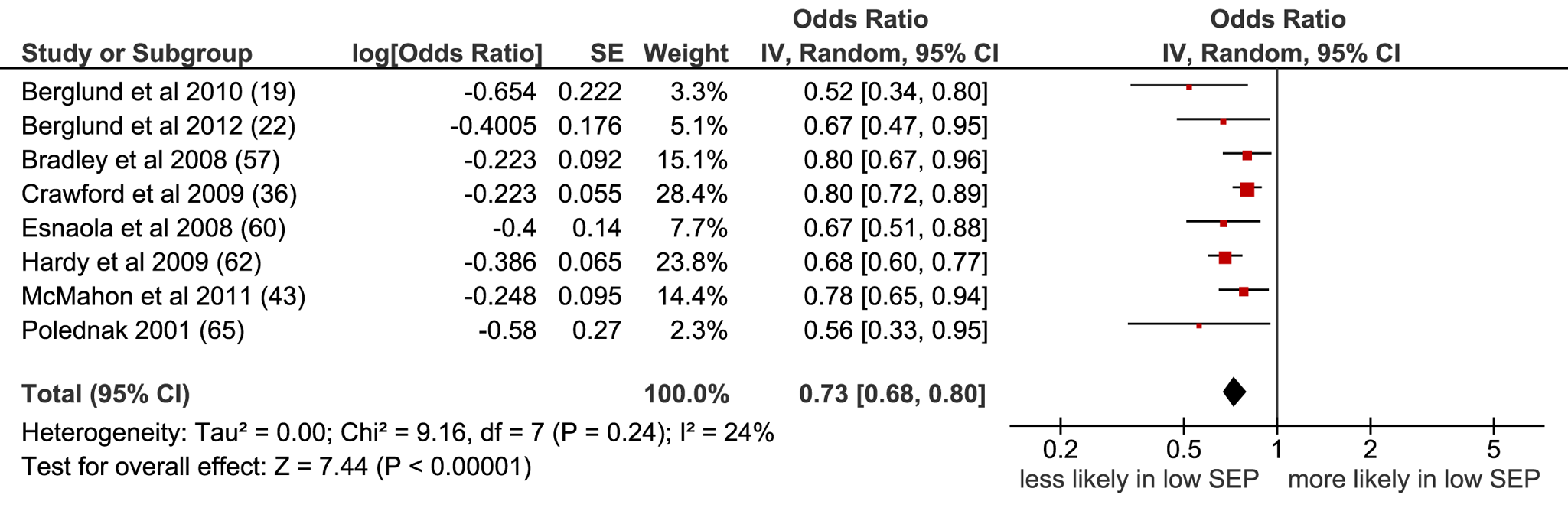

Supplement: Figure S2 — Meta-analysis of odds of receipt of surgery for NSCLC in low versus high SEP (non-overlapping populations). CI, confidence interval; OR, odds ratio; SE, standard error; SEP, socioeconomic position. (TIF) [file pmed.1001376.s002.tif]

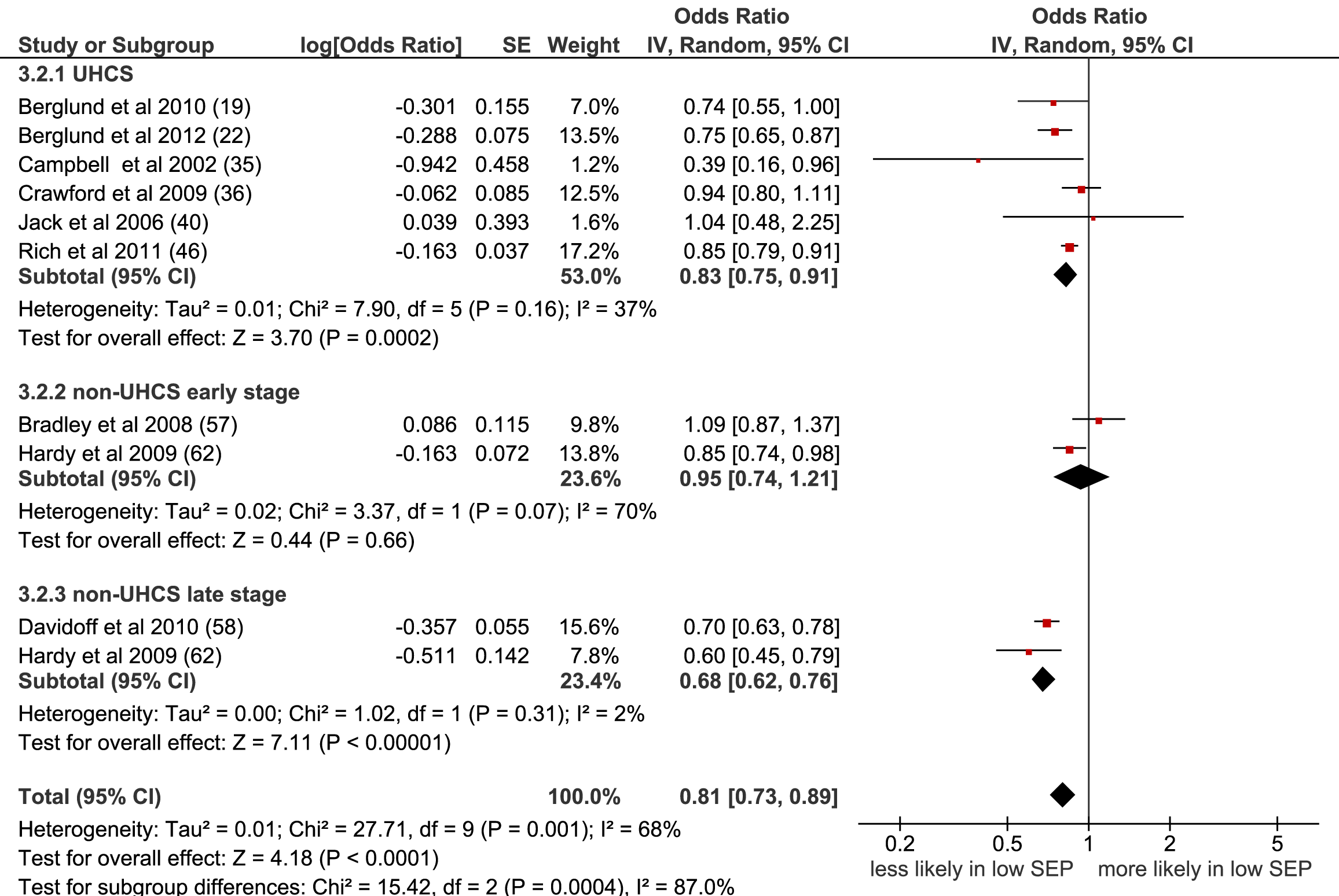

Supplement: Figure S3 — Meta-analysis of odds of receipt of chemotherapy in low versus high SEP (overlapping populations). CI, confidence interval; non-UHCS, non-universal health care system; OR, odds ratio; SE, standard error; SEP, socioeconomic position; UHCS, universal health care system. (TIF) [file pmed.1001376.s003.tif]

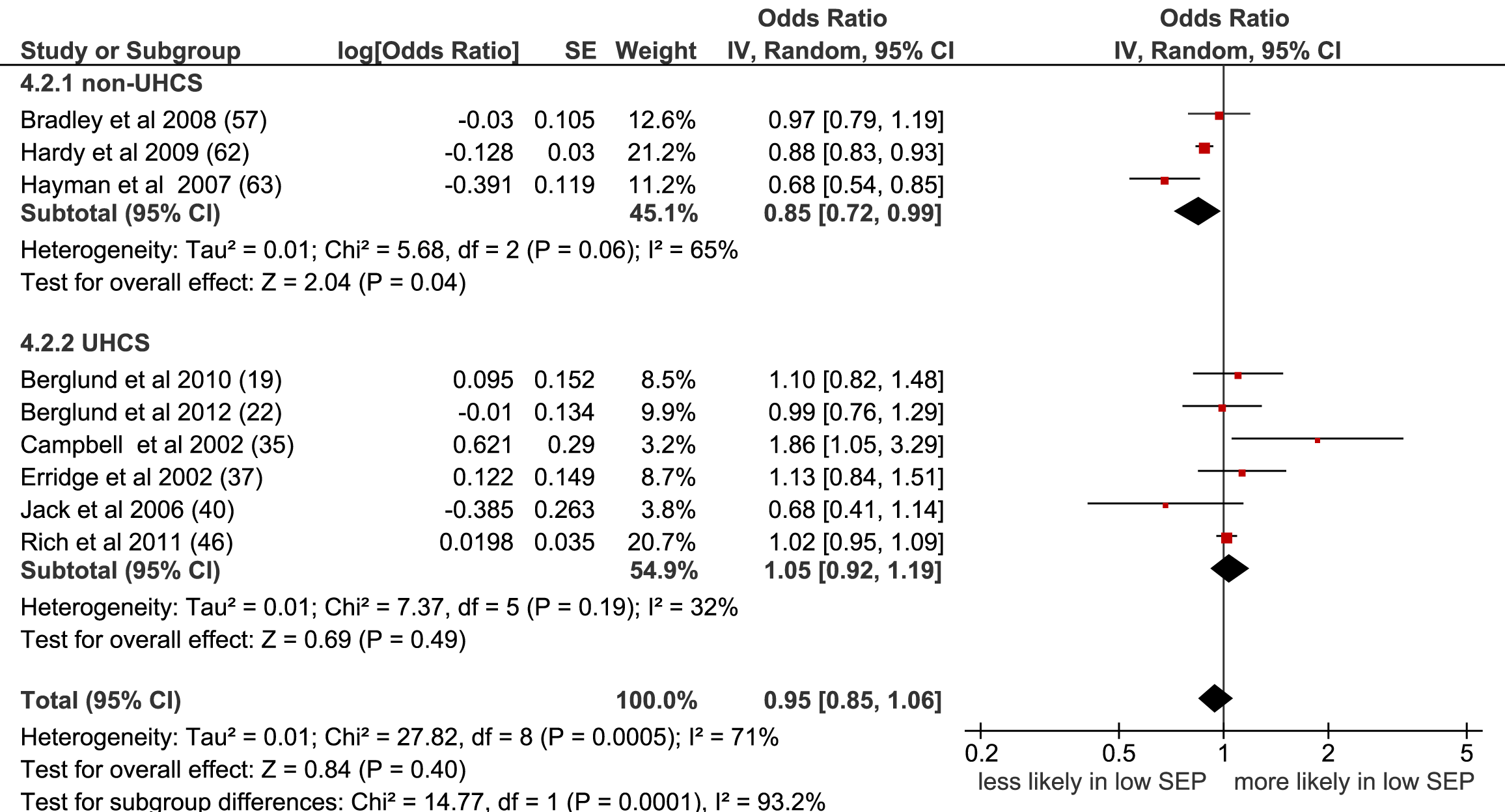

Supplement: Figure S4 — Meta-analysis of odds of receipt of radiotherapy in low versus high SEP (overlapping populations). CI = confidence interval, non-UHCS = non-universal health care system, OR = odds ratio, SE = standard error, SEP = socioeconomic position UHCS = universal health care system. (TIF) [file pmed.1001376.s004.tif]

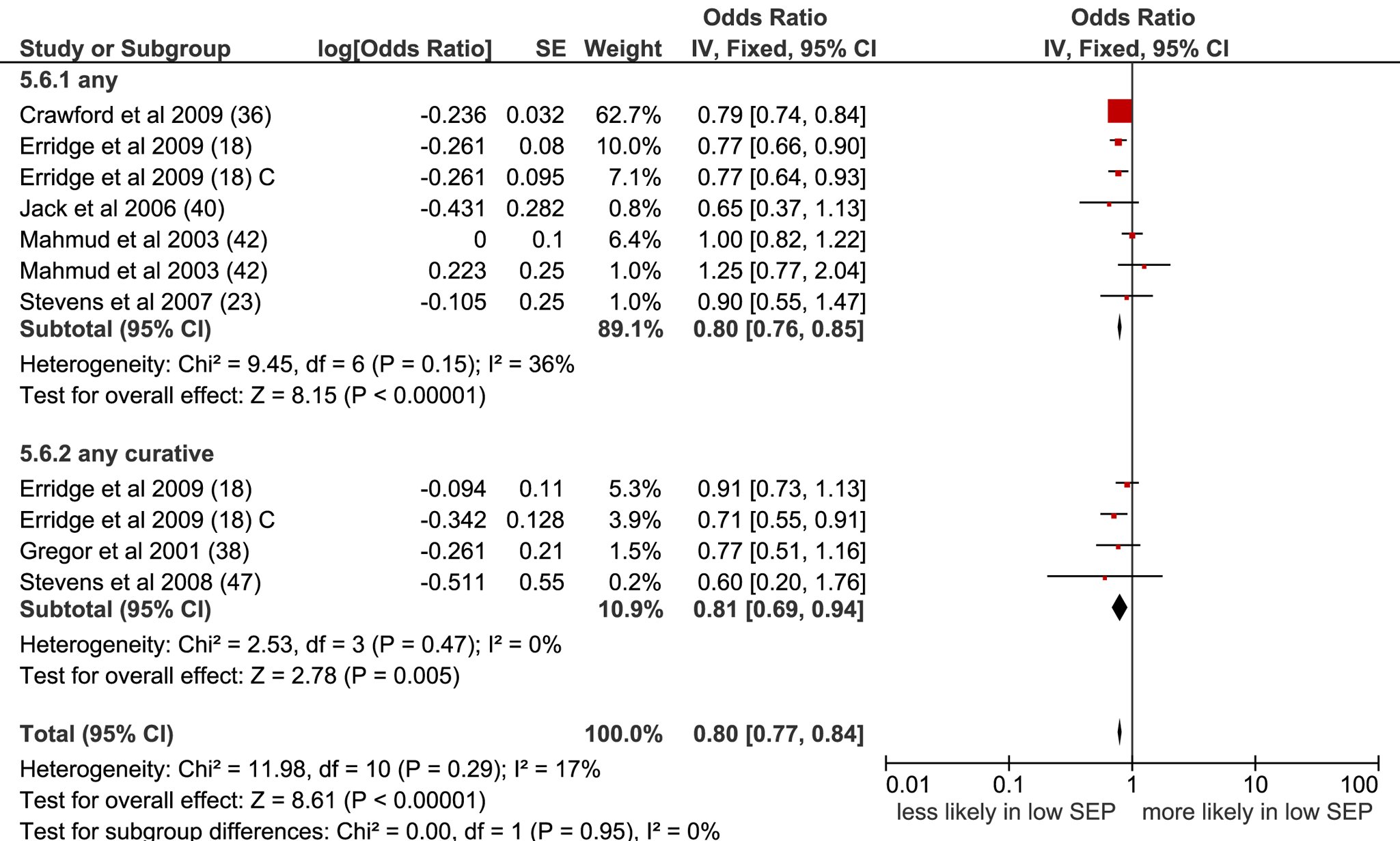

Supplement: Figure S5 — Sensitivity meta-analysis of odds of receipt unspecified treatment in low versus high SEP (overlapping populations). CI, confidence interval; OR, odds ratio; SE, standard error; SEP, socioeconomic position. (TIF) [file pmed.1001376.s005.tif]

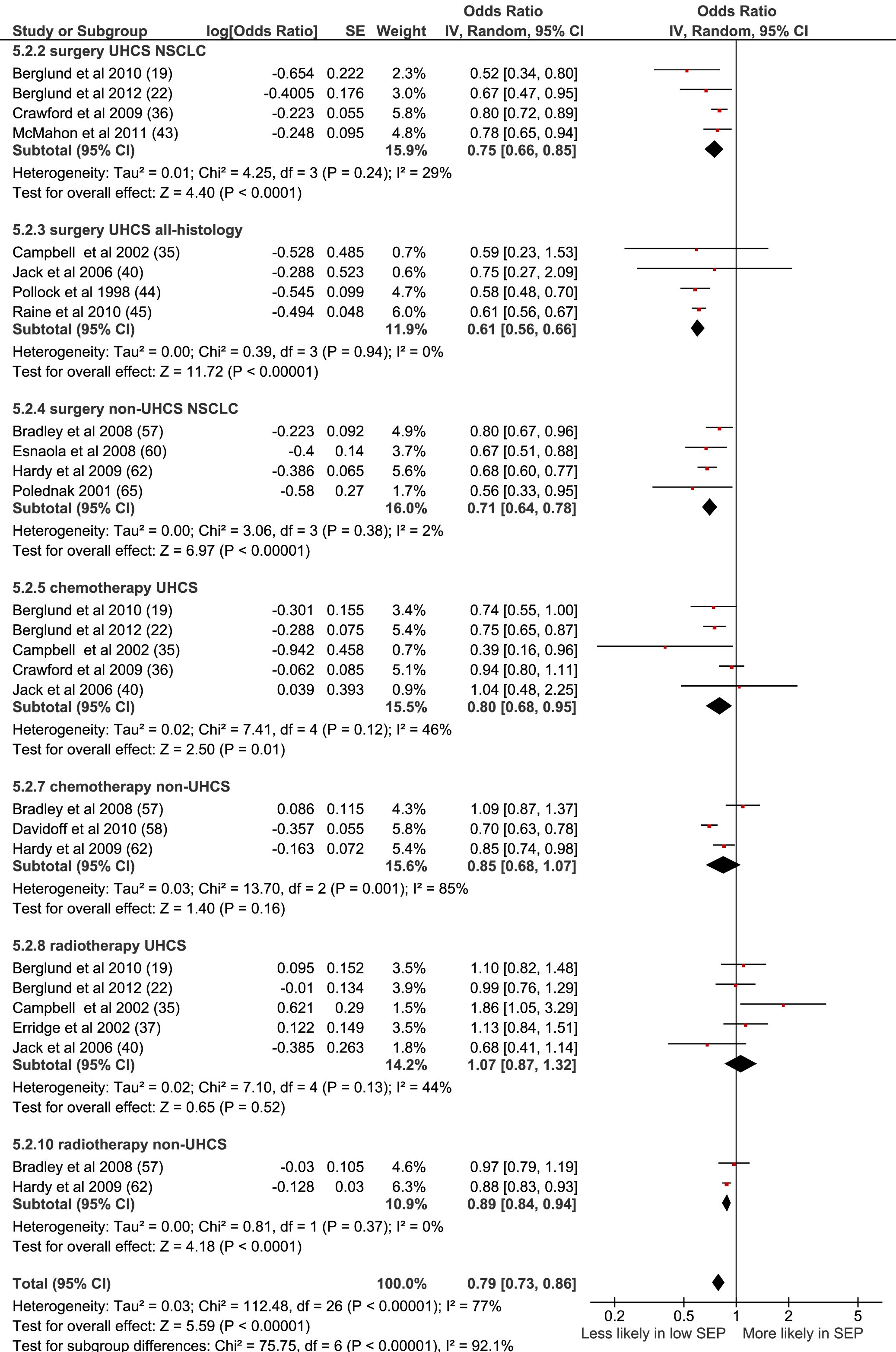

Supplement: Figure S6 — Meta-analysis of odds of receipt of any type of treatment in low versus high SEP. CI, confidence interval; non-UHCS, non-universal health care system; OR, odds ratio; SE, standard error; SEP, socioeconomic position; UHCS, universal health care system. (TIF) [file pmed.1001376.s006.tif]

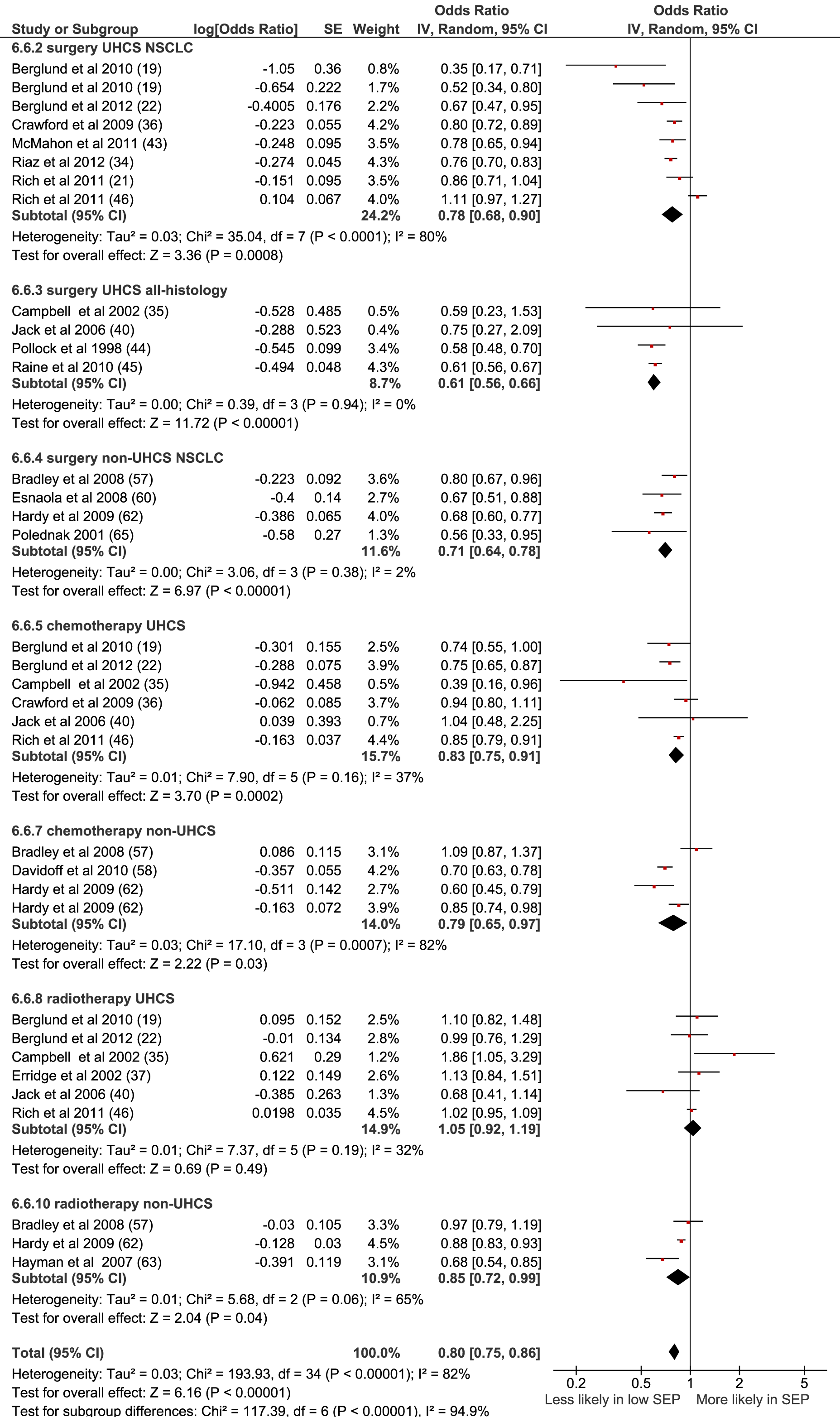

Supplement: Figure S7 — Meta-analysis of odds of receipt of any type of treatment in low versus high SEP (overlapping populations). CI, confidence interval; non-UHCS, non-universal health care system; OR, odds ratio; SE, standard error; SEP, socioeconomic position; UHCS, universal health care system. (TIF) [file pmed.1001376.s007.tif]

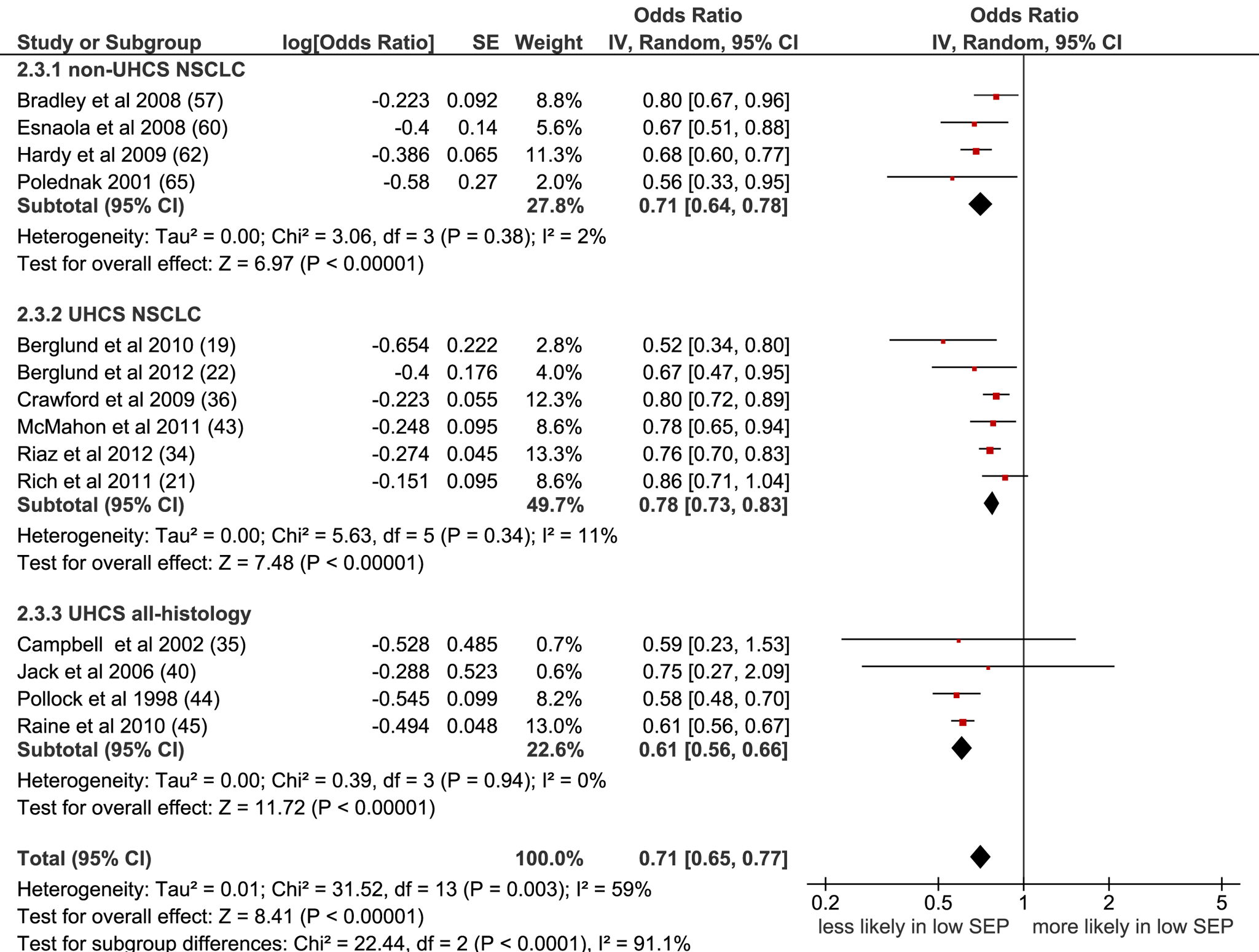

Supplement: Figure S8 — Meta-analysis of odds of receipt of surgery in low versus high SEP (partially-overlapping populations). CI, confidence interval; non-UHCS, non-universal health care system; OR, odds ratio; SE, standard error; SEP, socioeconomic position; UHCS, universal health care system. (TIF) [file pmed.1001376.s008.tif]

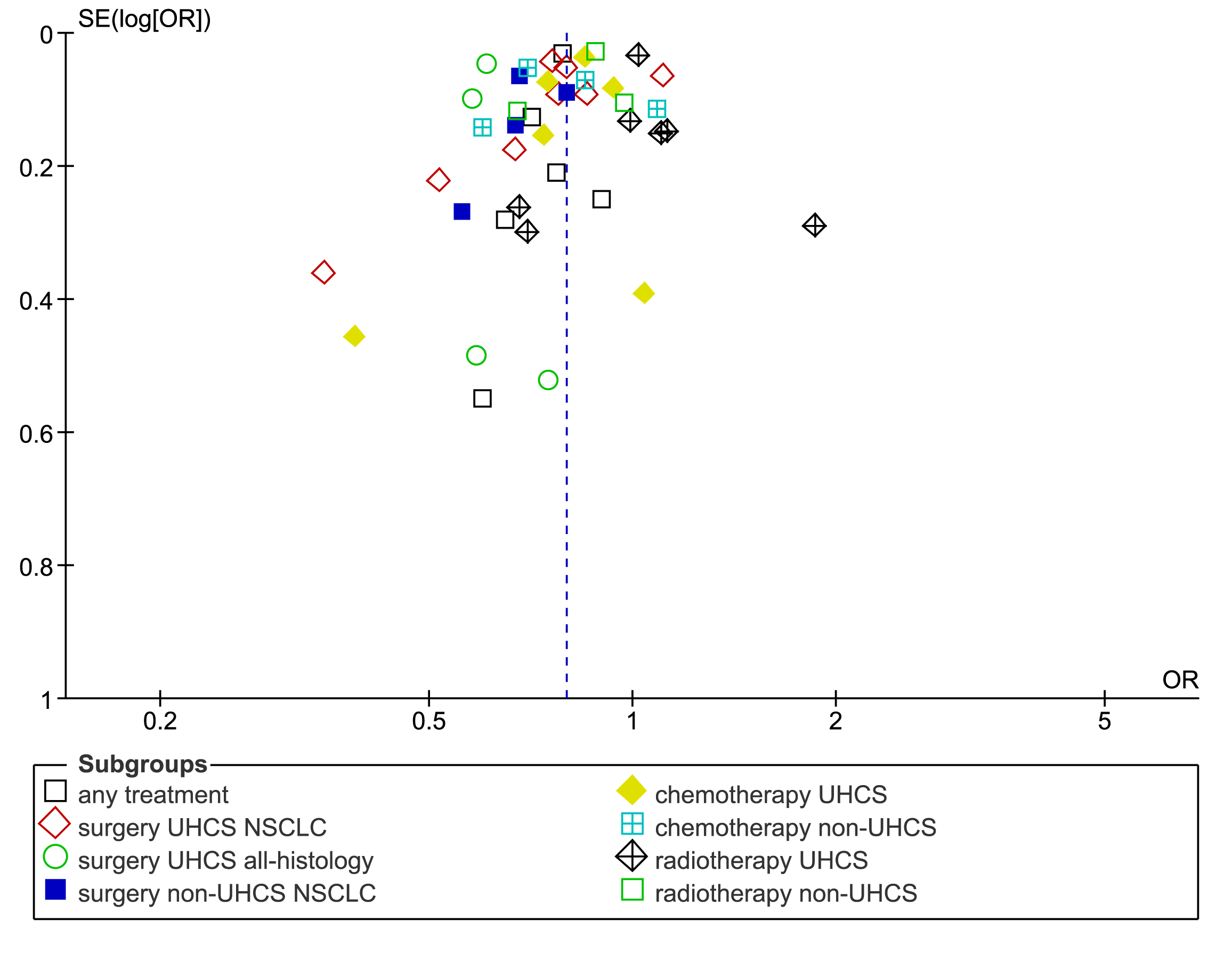

Supplement: Figure S9 — Funnel plot to assess publication bias. CI, confidence interval; non-UHCS, non-universal health care system; NSCLC, non-small cell lung cancer; UHCS, universal health care system. (TIF) [file pmed.1001376.s009.tif]
