# Supplementary material for: Socioeconomic Inequalities in Lung Cancer Treatment: Systematic Review and Meta-Analysis
Source: PLoS Med. 2013 Feb 5;10(2):e1001376. doi: 10.1371/journal.pmed.1001376 (PMC3564770; doi:10.1371/journal.pmed.1001376)
Supplement: Table S1 — Full search strategies (MEDLINE and EMBASE). (DOC) [file pmed.1001376.s010.doc]

**Table S1**

***Search strategies***

Medline search strategy 05/05/11

| **Search Term** | **Number Retrieved** |
| --- | --- |
| 1.       Lung Neoplasms/ di, ep, mo, pc, rt, su, | 59693 |
| 2.       Exp carcinoma, Non-Small-cell Lung/or exp Carcinoma, Small Cell/ | 37049 |
| 3.       Or/1-2 | 80591 |
| 4.       Social Class/ or Socio-economic Factors/ | 110317 |
| 5.       Socio-economic status.mp | 4098 |
| 6.       Education/ or exp Education, Continuing | 64913 |
| 7.       Income/cl, sn | 3109 |
| 8.       Exp Health Status/sn, td | 1046 |
| 9.       Exp Poverty/pc, sn, td | 2309 |
| 10.   Exp Social Class/ | 27097 |
| 11.   Socio-economic position.mp | 213 |
| 12.   Inequalities.mp | 5837 |
| 13.   Exp Social Environment/td | 137 |
| 14.   Social factors.mp | 4869 |
| 15.   Income.mp | 53934 |
| 16.   Exp Residence Characteristics/cl, sn | 3307 |
| 17.   Social Class.mp | 29869 |
| 18.   Education.mp | 463082 |
| 19.   Exp Health Status Disparities/ | 3380 |
| 20.   Inequities.mp | 1569 |
| 21.   Deprivation.mp | 51228 |
| 22.   Equity.mp | 5183 |
| 23.   Inequity.mp | 920 |
| 24.   Insurance status.mp | 1700 |
| 25.   Or/ 4-24 | 650350 |
| 26.   surgery.mp | 660227 |
| 27.   treatment.mp | 255751 |
| 28.   exp Health Services Accessibility/cl, og, st, sn, td, ut | 16904 |
| 29.   exp Healthcare Disparities/ | 3400 |
| 30.   treatment disparities.mp | 69 |
| 31.   exp “Delivery of Health Care”/ | 662295 |
| 32.   exp Primary Health Care/sn, td, ut | 6839 |
| 33.   exp Drug Therapy/ | 921829 |
| 34.   Chemotherapy.mp or | 236003 |
| 35.   Radiotherapy, Adjuvant/ or Radiotherapy/ | 45648 |
| 36.   Radiotherapy.mp | 145755 |
| 37.   Accessibility.mp | 56793 |
| 38.   Access.mp | 123272 |
| 39.   Pattern$.mp | 767175 |
| 40.   Palliative care/ or Patient care/ or Primary Health care/ | 83405 |
| 41.   Care.mp | 1154474 |
| 42.   Investigation.mp | 282065 |
| 43.   Exp “Quality of Health Care”/st, sn, td, ut | 99809 |
| 44.   Exp Patient Selection/ or exp Eligibility Determination/or exp Medicaid/ | 60372 |
| 45.   Exp “Referral and Consultation”/ st, sn, td, ut | 9243 |
| 46.   Receipt.mp or exp “Patient Acceptance of Health Care”/ | 137892 |
| 47.   Provision.mp | 33164 |
| 48.   Attendance.mp | 11676 |
| 49.   Or/26-48 | 5529748 |
| 50.   3 and 25 and 49 | 484 |
| 51.   News.pt | 130842 |
| 52.   Comment.pt | 438297 |
| 53.   Letter.pt | 712489 |
| 54.   Review pt | 1600963 |
| 55.   Editorial.pt | 274165 |
| 56.   50 not (or/51-55) | 398 |

Embase search strategy 05/05/11

| **Search Term** | **Number Retrieved** |
| --- | --- |
| 1.       Exp lung cancer/ di, dm, dt, ep, rt, rh, su, th | 71888 |
| 2.       Exp LUNG CARCINOMA/ di, dm, dt, ep, rt, rh, su, th | 43143 |
| 3.       Exp lung non-Small-cell cancer/ di, dm, dt, ep, rt, rh, su, th | 25650 |
| 4.       exp small cell carcinoma/ di, dm, dt, ep, rt, rh, su, th | 4284 |
| 5.       Or/1-4 | 75298 |
| 6.       Social Class/ or Socio-economic Factors/ | 105019 |
| 7.       Socio-economic status.mp | 5032 |
| 8.       Education/ or exp Education, Continuing | 264216 |
| 9.       Socio-economic position.mp | 254 |
| 10.   Social factors.mp | 5735 |
| 11.   Income.mp | 60981 |
| 12.   Social Class.mp | 24420 |
| 13.   Education.mp | 463082 |
| 14.   Exp LOWEST INCOME GROUP/ or exp INCOME/ | 54541 |
| 15.   Exp Poverty/ | 23046 |
| 16.   Inequality.mp | 7552 |
| 17.   Inequalities.mp | 6594 |
| 18.   Exp Social Environment/ | 224660 |
| 19.   Exp demography/ | 114434 |
| 20.   Exp health disparity/ | 2317 |
| 21.   Exp Health insurance/ or exp socioeconomics/ or exp Social status/ | 308865 |
| 22.   Inequity.mp | 1106 |
| 23.   Equity.mp | 5975 |
| 24.   Exp CULTURAL DEPRIVATION/ or deprivation.mp | 53312 |
| 25.   Or/ 6-24 | 1301033 |
| 26.   surgery.mp | 1211103 |
| 27.   treatment.mp | 3334976 |
| 28.   treatment disparities.mp | 92 |
| 29.   exp “Delivery of Health Care”/ | 1383093 |
| 30.   exp Drug Therapy/ | 1219237 |
| 31.   Chemotherapy.mp or | 357346 |
| 32.   Radiotherapy, Adjuvant/ or Radiotherapy/ | 68286 |
| 33.   Radiotherapy.mp | 202245 |
| 34.   HEALTH CARE ACCESS/ | 27526 |
| 35.   Access.mp | 176386 |
| 36.   Care.mp | 1749819 |
| 37.   Pattern$.mp | 824237 |
| 38.   Health service/ or health care policy/ or equity.mp or health care/ or health care delivery | 332536 |
| 39.   Quality.mp or HEALTH CARE QUALITY/ | 731294 |
| 40.   Health care utilization/ | 28472 |
| 41.   Provision.mp | 39964 |
| 42.   Attendance.mp | 14211 |
| 43.   Receipt.mp | 7755 |
| 44.   Terminal care/ | 18463 |
| 45.   Or/26-44 | 7347896 |
| 46.   5 and 25 and 45 | 1708 |
| 47.   Letter.pt | 726344 |
| 48.   Editorial.pt | 370622 |
| 49.   Note.pt | 440574 |
| 50.   Review.pt | 1692350 |
| 51.   46 not (or/47-50) | 1208 |
| 52.   51 and article.pt | 1080 |
