# Supplementary material for: Socioeconomic Inequalities in Lung Cancer Treatment: Systematic Review and Meta-Analysis
Source: PLoS Med. 2013 Feb 5;10(2):e1001376. doi: 10.1371/journal.pmed.1001376 (PMC3564770; doi:10.1371/journal.pmed.1001376)
Supplement: Text S2 — Protocol. (DOC) [file pmed.1001376.s012.doc]

**Protocol**

**Socio-economic inequalities in lung cancer treatment: systematic review and meta-analysis**

**Lynne F Forrest**^1^ MSc (Lynne.Forrest1@ncl.ac.uk)

^1^Institute of Health and Society, Newcastle University, Newcastle upon Tyne, UK

**Summary**

***Background***

Intervention-generated inequalities are health inequalities that result from the way that health interventions are organised and delivered and there is some evidence to suggest that intervention-generated inequalities in care may occur for some common cancers. Although the incidence and outcome of lung cancer varies with socio-economic position (SEP), it is not known whether inequalities in treatment occur and, if they do, how these might contribute to inequalities in outcome.

***Review objectives***

To summarise the existing literature and assess whether there are socio-economic differentials in receipt of treatment for lung cancer

***Population***

Adults who have a primary diagnosis of lung cancer (ICD10 C33 and C34), participating in studies published in a peer-reviewed journal up to 2011, and where the relevant outcome is analysed according to a measure of socio-economic position (including an individual or area-based measure of socio-economic status (SEP), deprivation, income, or education).

***Interventions and comparators:***

Receipt of any curative or palliative treatment for lung cancer including surgery, chemotherapy and radiotherapy compared to not receiving surgery, chemotherapy or radiotherapy

***Outcomes:***

i) Rates of treatment; or ii) Odds of receiving treatment; looking at low compared to high SEP or trends by socio-economic strata

***Study design:***

Cohort, observational studies conducting appropriate univariable or multivariable analyses

**Background**

***Lung cancer***

Worldwide, lung cancer is the most common cancer. In the UK it is the second most common cancer for incidence overall (the second most common for men and third most common for women) , as well as the most common cause of cancer mortality . Less than 10% of those diagnosed survive for 5 years.

Lung cancers are classified into small cell (SCLC) and non-small cell (NSCLC) cancers, with NSCLC accounting for 80% of lung cancers. NSCLC can be further divided into squamous cell carcinomas, adenocarcinomas and large cell carcinomas . NICE guidelines recommend radical surgery (pneumonectomy or lobectomy) for stage I or II NSCLC. Chemotherapy and radical radiotherapy are recommended for stage IIIa, with chemotherapy for stage IIIb and good performance-status stage IV lung cancer patients. Radiotherapy may be given as a palliative option for stage IV patients with poor performance status. Intervention with surgery, chemotherapy or radiotherapy has been shown to improve survival .

The incidence and outcome rates of lung cancer vary with socio-economic position (SEP), with incidence and mortality rates 2-3 times higher in the more deprived, within the UK . A strong deprivation gradient for incidence and mortality is also seen worldwide. However, it is not known whether inequalities in investigation and treatment occur and, if so, how these might contribute to inequalities in outcome.

***Intervention-generated inequalities***

Intervention-generated inequalities are health inequalities that result from the way that health interventions are organised and delivered so that although overall health may improve as the result of an intervention, differences in access to the intervention, differential uptake and delays in uptake might result in inequalities in outcome. Inequalities are likely to occur at many different stages of intervention pathways and act in a cumulative way. It is also likely that intervention-generated inequalities contribute to overall socio-economic inequalities in morbidity and mortality, although this has not been conclusively demonstrated .

Inequalities in cancer care within the UK have been noted and the NHS Cancer Plan in 2000 pledged to reduce cancer mortality, reduce delay in diagnosis and treatment and increase survival whilst acting to reduce inequalities . More recently the National Cancer Equality Initiative has been set up to address some of these issues . This is an important task as, in a 2006 review that summarised a decade of research on the association between socio-economic status and cancer survival, the authors suggested that socio-economic differences in ‘access to optimal treatment’ might at least partially explain survival differences.

Inequalities in access to cancer care have been shown in individual studies for a number of cancers and in a non-systematic review for colorectal cancer but the evidence is inconclusive and there has been no systematic review of the evidence to demonstrate if such inequalities in access to care exist for lung cancer.

**Review Objectives**

To summarise the existing literature and assess whether there are socio-economic differentials in receipt of treatment for lung cancer

**Methods**

***Search strategy***

Systematic methods will be used to identify relevant studies, assess study eligibility for inclusion and evaluate study quality. A search will be undertaken to locate all studies published up to May 2011 examining care and treatment for lung cancer associated with socio-economic status. One researcher (LF) will develop the search strategy with support from her supervisors, which will then be refined with the help of a medical librarian and used to search the online databases of MEDLINE, EMBASE and Scopus. Slightly different strategies will be required for each database (for example MEDLINE recognises the MESH term Lung Neoplasms/ whereas EMBASE does not and uses Lung cancer/. See pp6-8 of this protocol for draft MEDLINE and EMBASE search strategies). Additional studies will be identified by reviewing the reference lists of relevant studies identified from the search and by using a forward citation search to identify more recent studies that have cited an older, relevant study. EndNote software will be used to manage the references.

***Study Eligibility***

Cohort studies of adult participants who have a primary diagnosis of lung cancer (small-cell lung cancer or non-small-cell lung cancer - ICD10 C33 or C34), published in a peer-reviewed journal up to 2011, and where the outcome is receipt of care or treatment (measured by rates or odds of receiving care/treatment) and where the outcome is analysed by a measure of socio-economic position (such as an individual or area-based measure of SEP, deprivation, income or education) will be eligible for inclusion. Receipt of any curative or palliative treatment for lung cancer including surgery, chemotherapy and radiotherapy will be considered.

Preliminary independent screening of the titles and abstracts obtained from the database searches will be carried out by two researchers (LF and HW). Initial screening of titles will be carried out to remove obviously irrelevant papers. However, from a preliminary scoping review by LF, the early pilot searches recovered studies that, although they conducted analyses by SEP, did not always mention this in the abstract or title. Therefore, in the title search, any titles that refer to surgery, chemotherapy and radiotherapy uptake for lung cancer will be retained. Papers that look at disparities in cancer survival/mortality will also be included as further checking of the abstract is required to see if inequalities in access to treatment are also examined.

Selected abstracts will then be screened and a subset of studies will be selected for further review and the full article obtained. Abstracts that refer to socio-economic inequalities in receipt of care/treatment will be retained. Abstracts that refer to racial, ethnic, geographical, sex and age-related disparities in treatment as well as disparities by insurance type will also be retained as often these papers also look at SEP, even if this is not mentioned in the abstract. Papers that look at delay will not be included. Two researchers (LF and HW) will then independently assess the selected full papers for eligibility according to the study-eligibility criteria detailed above. Any disagreements at any of the screening stages will be resolved by discussion between the two reviewers in the first instance. If agreement cannot be reached, then a third reviewer (JA or MW) will independently review the title, abstract or full paper, as appropriate, and a majority decision will be taken on inclusion/exclusion.

***Data Extraction***

Data extraction will be carried out by LF and HW using a pro-forma to be developed by LF for this purpose. Data relating to study authors, journal, study design, year of study, data source, number of participants, years of diagnosis, measure of SEP, confounding variables included in the analysis (such as age, sex, stage, co-morbidities, cancer type/site, vital/performance status, marital status, smoking status, cancer network, health board, hospital, emergency or elective treatment, distance from hospital/travel time, ethnicity, insurance status), type of treatment received (any, surgery, chemotherapy, radiotherapy), statistical tests carries out, outcome measures (treatment rates or odds of treatment), comparator used, significance (p values), precision (confidence intervals), other variables that were significant; will be recorded.

There is evidence to suggest that insurance status is an important factor relating to access to lung cancer care in the US system but is less relevant or rarely measured in the UK and Europe. Therefore studies will be split into three categories: those carried out in a healthcare system free at the point of access (similar to the UK); those based on an insurance system (similar to the USA); those that include a mixture of free care and social insurance-based payment (some European systems).

***Study Quality***

Study quality will be appraised using criteria based on the SIGN guidelines and the STROBE (Strengthening the Reporting of Observational Studies in Epidemiology) guidelines that contain a checklist of 22 items that should be included in cohort studies. Although the STROBE guidelines are a checklist measure of good reporting rather than ‘an instrument to evaluate the quality of observational research’ (ref), a number of other lung cancer systematic reviews use a similar scale for quality ascertainment .

The quality of reporting on the following criteria will be assessed: study design, size, setting, dates, data sources, eligibility criteria, number of participants potentially eligible, number actually included, number analysed, missing/incomplete data reported, variables included (in terms of outcome, exposure, predictors, confounders), type of statistical analysis carried out, unadjusted and adjusted estimates reported, precision (confidence intervals), significance (p values) given, limitations of the study, potential bias addressed, external validity of results and funding source.

***Statistical analysis***

Meta-analysis will be considered if there are sufficient studies available with suitable data. If it is not possible to conduct a meta-analysis, due to the heterogeneity of the studies, then Harvest Plot methodology will be considered. This is a method that has been devised for synthesising evidence from studies looking at the differential effects of interventions, where meta-analysis is not suitable . Meta-regression may also be considered if there are sufficient studies with similar variables available that might enable combined analysis of factors associated with combined outcomes.

**References**
