# Supplementary material for: Socioeconomic Inequalities in Lung Cancer Treatment: Systematic Review and Meta-Analysis
Source: PLoS Med. 2013 Feb 5;10(2):e1001376. doi: 10.1371/journal.pmed.1001376 (PMC3564770; doi:10.1371/journal.pmed.1001376)
Supplement: Text S3 — Quality score checklist. (DOC) [file pmed.1001376.s013.doc]

**Text S3**

Quality Score

| Quality Score | Characteristics of studies |
| --- | --- |
| 6 | Multi-variable analysis. Population-based sample. Good internal validity/reporting/confounding |
| 5 | Multi-variable analysis. Selective population. Good internal validity/reporting/confounding |
| 4 | Multi-variable analysis. Population-based sample. Some issues with internal validity/reporting/confounding (eg good internal validity/reporting but stage not included OR less good internal validity/reporting and stage included) |
| 3 | Multi-variable analysis. Selective population. Some issues with internal validity/reporting/confounding (eg good internal validity/reporting but stage not included OR less good internal validity/reporting and stage included) |
| 2 | Univariable analysis. Good internal validity/reporting OR multivariable analysis but only univariable results reported or no CIs/ different comparator/ stratified by other variable |
| 1 | Univariable analysis. Poor internal validity/reporting OR multivariable analysis but results for SEP not shown or errors in data |

**Quality checklist used to derive quality scores**

***Screening questions:***

Does the study conduct **multivariable** analysis and report **adjusted** odds ratios/rates?

Yes – consider for meta-analysis

No – consider for narrative review

What **population** is included?

- 1. Population-based sample: eg total local or national lung cancer population from a cancer Registry
  2. Selective population: eg. hospital population from Hospital Episode Statistics (HES) data or similar, or from Registry linked to Medicare records (USA) or incomplete audit data

**Section 1: Internal validity**

1. Appropriate, valid and reliable measure of SEP is used (e.g. IMD, Carstairs, Townsend, similar local index, income, poverty level, education)
   1. Yes - individual standard measure used 5
   2. Yes - area-based standard measure used 3
   3. Standard measure used but presented as average for PCT/health authority 1
   4. Non-standard measure used/ measure not reported 0
2. SEP categorised as
   1. Continuous/Deciles 5
   2. Quintiles/ Quartiles 3
   3. Tertiles/Dichotimised 1
   4. Unknown/ not reported 0
3. SEP expressed as an OR to
   1. Two decimal places 5
   2. One decimal place 3
   3. Whole number/ expressed as rate 1
   4. unknown/not reported 0
4. Outcome measures are valid and reliable
   1. Yes - care/ treatment details obtained from Registry, HES or similar system 3
   2. Yes - care/treatment details obtained from audit or hospital records 3
   3. Care treatment details obtained from survey/questionnaire/other 1
   4. No/unknown/not reported 0

**Good: 14-18 OK: 13-11 Poor: 0-10**

**Section 2: External validity**

1. Population
   1. Study uses multiple Registry or national population(s) or similar 3
   2. Study uses regional population from Registry or HES or similar 2
   3. Study uses only small subset of population (some health boards/PCTs/areas) 1
   4. Study uses small, random sample from Registry or hospital data/ other 1
   5. Population not stated 0
2. Population date and time period
   1. Multiple years of diagnosis, some post-2000 3
   2. Multiple years of diagnosis, all pre-2000 2
   3. Single year of diagnosis, post -2000 2
   4. Single year of diagnosis, pre-2000 1

**Good: 4-6 Poor: 0-3**

**Section 3: Reporting of Study**

1. Outcome measures are clearly defined/reported
   1. Yes 1
   2. No 0
2. Outcome measure reported
   1. Rates/odds of treatment compared with no treatment 2
   2. Rates/odds of NOT receiving treatment compared to receiving treatment 2
   3. Rates/odds of treatment compared with other care 1
   4. Rates/odds of treatment stratified by other variable (sex, race etc) 1
   5. Results presented in some other way 0
3. Number initially eligible/ number excluded reported
   1. Eligible/excluded/included all reported or able to be calculated 2
   2. Number included reported only 1
   3. Numbers not reported 0
4. Inclusion/ exclusion criteria detailed
   1. Yes 1
   2. No 0
5. Numbers receiving treatment
   1. Numerator and denominator populations clearly documented 2
   2. Numbers calculable from details given but not clearly specified 1
   3. Numbers not specified 0
   4. Numbers do not add up correctly and need to be checked with authors 0
6. Other variables that are significant in analysis reported
   1. Yes 2
   2. Yes, but results for only some presented 1
   3. No 0
7. Death Certificate Only (DCO) excluded
   1. Yes 1
   2. Not applicable (if using HES type data) 1
   3. No/ not reported 0
8. Confidence interval reported
   1. Yes 1
   2. No 0
9. P value reported
   1. Overall p value/ p for trend 2
   2. Individual p values 1
   3. Not reported 0

**Good: 11-14 OK: 7-10 Poor: 0-6**

**Section 4: Confounding**

1. Multivariable analysis - other important confounders included
   1. Age and sex 3
   2. Age or Sex 2
   3. Univariable analysis only reported 1
   4. Descriptive only/ no analysis 0
2. Results stratified by stage
   1. Yes, and only eligible- stage patients used for denominator 3
   2. Yes, but all-stage patients used for denominator 2
   3. No, but stage included as a confounder 2
   4. No 0
3. Results stratified by histology
   1. Yes, and only histologically-verified cases included 3
   2. Yes, but clinically diagnosed and histologically-unknown cases included 2
   3. No, but histology included as a confounder 2
   4. No 0
4. Other relevant confounders included
   1. Co-morbidity/ performance status 2
   2. Trust/ health board/ hospital/area 1
   3. No 0

**Good: 7-11 (must include age, sex, stage and histology)**

**OK: 3-5 (must include age, sex)**

**Poor: 0-2 (univariable analysis)**
